# Ozone- and Hydroxyl Radical-Mediated Oxidation of Pharmaceutical Compounds Using Ni-Doped Sb–SnO_2_ Anodes: Degradation Kinetics and Transformation Products

**DOI:** 10.1021/acsestengg.2c00337

**Published:** 2023-01-26

**Authors:** Yi Zhang, Lei Guo, Michael R. Hoffmann

**Affiliations:** † Linde Laboratories, California Institute of Technology, Pasadena, California91125, United States; ‡ Department of Civil Engineering, University of Arkansas, Fayetteville, Arkansas72701, United States

**Keywords:** ozone- and hydroxyl radical-mediated oxidation, Ni-doped Sb−SnO_2_ anodes, pharmaceuticals, degradation kinetics, transformation products

## Abstract

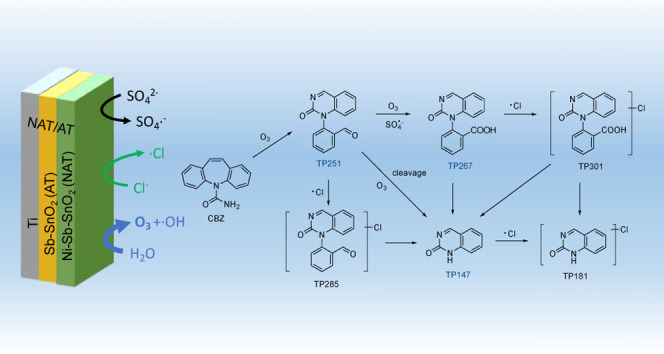

Electrochemical oxidation provides a versatile technique for treating wastewater streams onsite. We previously reported that a two-layer heterojunction Ni–Sb–SnO_2_ anode (NAT/AT) can produce both ozone (O_3_) and hydroxyl radical (^•^OH). In this study, we explore further the applicability of NAT/AT anodes for oxidizing pharmaceutical compounds using carbamazepine (CBZ) and fluconazole (FCZ) as model probe compounds. Details of the oxidation reaction kinetics and subsequent reaction products are investigated in the absence and presence of chloride (Cl^–^) and sulfate (SO_4_^2–^). In all cases, faster or comparable degradation kinetics of CBZ and FCZ are achieved using the double-layered NAT/AT anode coupled with a stainless steel (SS) cathode in direct comparison to an identical setup using a boron-doped diamond anode. Production of O_3_ on NAT/AT enhances the elimination of both parent compounds and their transformation products (TPs). Very fast CBZ degradation is observed during NAT/AT-SS electrolysis in both NaClO_4_ and NaCl electrolytes. However, more reaction products are identified in the presence of Cl^–^ than ClO_4_^–^ (23 TPs vs 6). Rapid removal of FCZ is observed in NaClO_4_, while the degradation rate is retarded in NaCl depending on the [Cl^–^]. In SO_4_^2–^-containing electrolytes, altered reaction pathways and transformation product distributions are observed due to sulfate radical generation. SO_4_^·–^ oxidation produces fewer hydroxylated products and promotes the oxidation of aldehydes to carboxylic acids. Similar trend in treatment performance is observed in mixtures of CBZ and FCZ with other pharmaceutical compounds in latrine wastewater and secondary WWTP effluent.

## Introduction

Pharmaceuticals represent an important group of emerging environmental contaminants due to excretion of ingested medicines in urine and feces and through the intentional disposal of unused or expired medicines.^1^ Depending on the specific drug types, more than 90% of consumed pharmaceuticals can be excreted unmetabolized. Thus, residual pharmaceutical products have been detected in virtually all environmental waters including groundwater, surface water, and wastewater treatment plant (WWTP) influents and effluents.^1−4^ This level of discharge of untreated or partially treated pharmaceutical products is problematic for aquatic systems and drinking water supplies. While traditional WWTPs are inadequate for degrading many commonly used pharmaceutical compounds,^5−7^ advanced oxidation processes (AOPs) involving reactive species such as ozone (O_3_), hydroxyl radicals (^•^OH), and free reactive chlorine provide an attractive alternative for compound degradation.^8−10^ Meanwhile, transformation products of pharmaceuticals also are an area of increasing research interest. The identification of specific transformation products allows us to further understand their ultimate environmental fates, including the formation of transformation products that are more toxic than their parent compounds.^1,11−13^

Electrochemical oxidation (EO) is applied in decentralized and small-scale wastewater treatment systems when proceeded by a biological pretreatment step.^14,15^ The composition of the electro-active anode materials is a key factor in determining energy consumption and electrolytic treatment efficiency. Ideal nonactive electrodes suitable for wastewater treatment that promote complete oxidation of organic pollutants include boron-doped diamond (BDD) and antimony-doped tin oxide (AT: Sb–SnO_2_) anodes.^14,16,17^ While large-scale application of BDD electrodes is hindered by high manufacturing costs, typical AT electrodes offer a less expensive option due to the absence of platinum-group metal components and lower production costs. Moreover, when nickel is co-doped with AT (NAT: Ni–Sb–SnO_2_), the NAT anodes produce ozone,^18,19^ which provides an additional anodic oxidation pathway.

Electrochemical oxidation has been investigated for various pharmaceutical compounds using dimensionally stable anodes (Ti/Pt/PbO_2_),^20^ mixed metal-oxide (Ti/Ir*_x_*Ta*_y_*O_2_/[Bi_2_O_3_]*_z_*[TiO_2_]_1–*z*_),^15^ and mostly BDD electrodes^20−22^ in pure electrolytes, latrine wastewater, and biologically treated hospital wastewater. However, degradation with Sb–SnO_2_-based anodes has not been well studied, and transformation product analysis is limited. In our previous study, a heterojunction Ni–Sb–SnO_2_ anode (NAT/AT) was prepared that simultaneously produces ozone (O_3_) and hydroxyl radical (^•^OH) at the anode.^23^ NAT/AT electrolysis was shown to be effective for treating a mixture of pharmaceutical compounds in toilet wastewater. An anodic O_3_ activation mechanism was proposed by Zhang et al. to explain the accelerated degradation kinetics of some aromatic compounds that are not highly reactive with O_3_ (e.g., ibuprofen, IBP, *k*_O_3_,IBP_ = 9.6 M^–1^ s^–1^).^8^ Since aromatic rings and/or N and S atoms with nonbonded electrons are ubiquitous in pharmaceutical compounds, NAT/AT electrolysis should prove to be effective for treating pharmaceuticals in waste effluents.

The primary goal of this study is to investigate both the kinetics and the possible mechanistic pathways for product formation via NAT/AT-SS electrolysis in undivided cells as a promising AOP for pharmaceutical compound treatment in contaminated water. Degradation kinetics and transformation products (TPs) formation are studied in detail for carbamazepine (CBZ) and fluconazole (FCZ). CBZ and FCZ are persistently found in natural water bodies around the world.^24,25^ They are also excellent probes for studying reaction mechanisms and evaluating treatment efficiencies due to their different biomolecular rate constants with O_3_ (*k*_O_3_,CBZ_ = 3.0 × 10^5^ M^–1^ s^–1^ vs *k*_O_3_,FCZ_ = 2.0 M^–1^ s^–1^).^8,26^ The added impact of electrochemical O_3_ production on the observed degradation kinetics and pathways in the NAT/AT system is compared to degradation using BDD electrodes as a reference anode material. The influence of pH and common ions present in wastewaters (Cl^–^ and SO_4_^2–^)^27,28^ is investigated. Kinetic models to predict overall reaction kinetics and removal efficiencies are presented. Furthermore, pharmaceutical degradation at the NAT/AT anode is evaluated in actual toilet wastewater and secondary effluent where the occurrence of these compounds is frequently reported.

## Materials and Methods

### Reagents and Wastewater

All pharmaceutical compounds, acridine, carbamazepine 10,11-epoxide, and 9-acridinecarboxylic acid standards were purchased from Sigma-Aldrich. Actual latrine wastewater was collected from a public electrochemical toilet prototype on the campus of Caltech (Pasadena, CA). Secondary effluent was obtained from Sanitation Districts of Los Angeles County (Whittier, CA) and stored in the dark at 4 °C for less than 1 month prior to use.

Stock solutions of individual pharmaceuticals were prepared at 20 μM in relevant electrolytes and stored under room temperature in the dark. Wastewater samples were filtered with 0.45 μm glass fiber membranes and amended with pharmaceuticals (2 μM) before treatment.

### Electrolysis Experiments

Two types of electrodes were used in this study. NAT/AT anodes were prepared by a dip-coating method as previously described.^23^ BDD electrodes were purchased from NeoCoat (Switzerland). All electrochemical tests were performed in an undivided electrolysis cell. A stainless steel cathode (6 cm^2^) was coupled in parallel to an NAT/AT or BDD anode (6 cm^2^) with a 5 mm separation. An Ag/AgCl/Sat. NaCl reference electrode (BASI, Inc.) was placed at the same spacing close to the anode. Electrolysis experiments were conducted in 25 mL solutions at a fixed current density of 10 mA/cm^2^ (specific surface area = 24 m^2^/m^3^).

Linear sweep voltammetry (LSV) was conducted using a Biologic VSP-300 potentiostat in relevant solutions using a scan rate of 0.05 V/s. For some experiments, solutions were buffered with 5 mM phosphate and pH was adjusted with perchloric acid (HClO_4_), hydrochloric acid (HCl), or sodium hydroxide (NaOH).

### Analytical Methods and Transformation Product Identification

Free chlorine concentrations were measured by DPD (*N*,*N*-diethyl-*p*-phenylenediamine) reagent (Hach DPD Method 10,102). Dissolved O_3_ produced by NAT/AT was quantified using the indigo method.^29^ Current efficiencies (η) for chlorine and O_3_ evolution were calculated using the equation
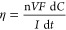
where *n* is the number of electrons required for 1 mole formation of Cl_2_ from Cl^–^ (*n* = 2) or O_3_ from O^2–^ (*n* = 6), *V* is the electrolyte volume (25 mL), *F* is Faraday constant (96,485 C/mol), and *I* is the current (A). Common wastewater ions (NH_4_^+^, Cl^–^, Na^+^, K^+^, and Mg^2+^) were quantified by ion chromatography.

Carbamazepine (CBZ), fluconazole (FCZ), and other pharmaceutical compounds were quantified using high-performance liquid chromatography coupled with a UV detector (HPLC-UV) and equipped with an XDB-C18 column (ZORBAX, 2.1 mm × 50 mm, 1.8 μm particles). CBZ and FCZ were monitored at 285 and 205 nm, respectively. Eluent flowed at 0.4 mL/min and consisted of acetonitrile (ACN) and water with 0.1% formic acid. A gradient was used to resolve peaks: 0 min, 5% ACN; 2 min, 5% ACN; 6 min, 95% ACN; 8 min, 95% ACN; 9 min, 5% ACN; 12 min, 5% ACN.

For transformation product identification, electrolysis of CBZ and FCZ was conducted at an elevated compound concentration (20 μM) and in solutions containing: NaClO_4_ (50 mM), Na_2_SO_4_ (5 mM) + NaClO_4_ (50 mM), NaCl (50 mM), or Na_2_SO_4_ (5 mM) + NaCl (50 mM). Products were analyzed using an ultrahigh-performance liquid chromatography system (Waters Acquity UPLC) coupled to a time-of-flight mass spectrometer (Waters Xevo GS-2 TOF) equipped with an Acquity BEH C18 column (2.1 mm × 50 mm, 1.7 μm particles). Eluent consisting of ACN and water with 0.1% formic acid flowed at 0.5 mL/min. The applied ACN/H_2_O gradient profile was as follows: 0 min, 5% ACN; 0.2 min, 5% ACN; 3.2 min, 95% ACN; 3.5 min, 95% ACN; 3.6 min, 5% ACN; 5 min, 5% ACN. Products were monitored under positive electrospray ionization (ESI+) in resolution mode (measurement accuracy better than 1 ppm RMS) with 0.2 kV capillary voltage. Other mass spectrometer conditions include: cone voltage 50 V, source offset 80 V, source temperature 120 °C, desolvation temperature 400 °C, cone gas 40 L/h, desolvation gas 800 L/h, 0.3 s scan time in continuum mode, collision energy 1.0 eV, and second acquisition channel collision energy scanned from 0 to 30 eV. A leucine lock-mass (*m*/*z* = 556.2771) was used to correct for accurate mass values.

Parent compounds and transformation products (TPs) were identified with the Masslynx software (Waters) and quantified with Quanlynx. TP chemical formulas were obtained using accurate mass determinations and known parent compound elemental compositions. Tentative structures were proposed based on fragmentation patterns, isotopic patterns (when chloride present), authentic standards (when available), and comparison to the literature.

### Theoretical Modeling

Kinetic modeling and prediction of pharmaceutical degradation kinetics were achieved using the chemical kinetics computational program, Kintecus 6.80.^30^ A total of 117 elementary reactions were considered with rate constants obtained from the literature. The model was evaluated in all electrolytes to examine the influence of salts on degradation kinetics (more details in Text S1 and Table S1).

## Results and Discussion

### Degradation Kinetics

Degradation was investigated in detail for carbamazepine (CBZ, 20 μM) and fluconazole (FCZ, 20 μM).

#### CBZ

**Figure 1:**
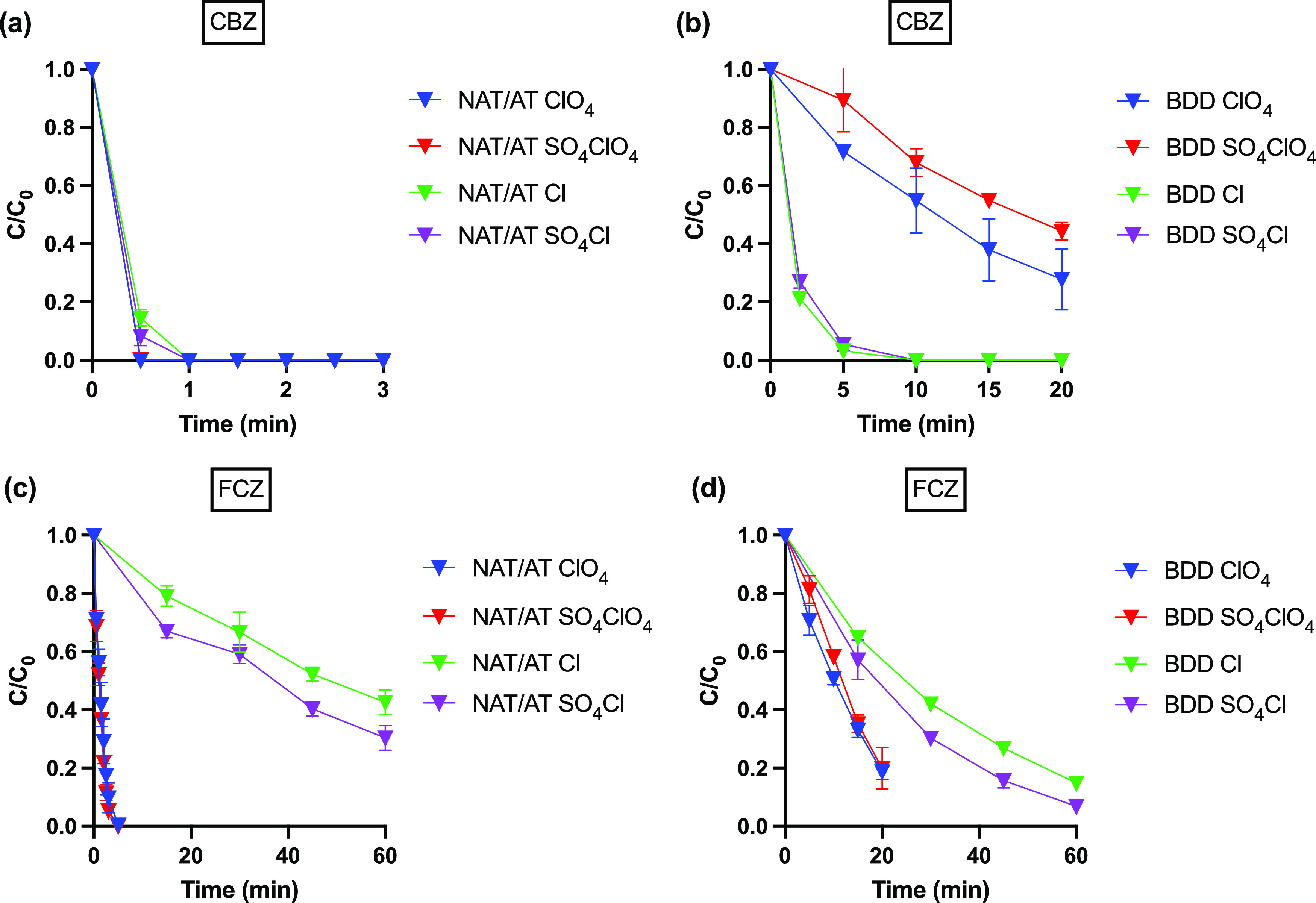
Electrochemical oxidation of carbamazepine (CBZ) by (a) NAT/AT and (b) BDD, and of fluconazole (FCZ) by (c) NAT/AT and (d) BDD in ClO_4_: 50 mM NaClO_4_, SO_4_ClO_4_: 5 mM Na_2_SO_4_ + 50 mM NaClO_4_, Cl: 50 mM NaCl, and SO_4_Cl: 5 mM Na_2_SO_4_ + 50 mM NaCl electrolytes.

Fast degradation of CBZ was observed using the NAT/AT anode (Figure 1a). For example, complete removal was achieved in less than 30 s in NaClO_4_ electrolytes, which appears to be due to direct oxidation by O_3_. Our kinetic model also predicted complete removal in 30 s (Figure S1a). When Cl^–^ is present in the system, O_3_ production is inhibited due to competition for active sites between Cl^–^ and OH^–^,^23^ although rapid removal is achieved with 100% removal in less than 1 min in NaCl electrolytes. Despite lower aqueous-phase O_3_ concentrations, a similar CBZ removal efficiency is achieved due to the formation of the chlorine radical anion Cl_2_^·–^ (*E*^0^ = 2.1 V_NHE_, estimated *k*_Cl_2_^·–^,CBZ_ = 2.6 × 10^9^ M^–1^ s^–1^).^11^ Prediction using the kinetic model also gave similar results taking into account the kinetics of the chlorine radical anion (Figure S1b).

Degradation of CBZ with BDD was slower in both electrolytes compared to that with NAT/AT due to the absence of O_3_ production (Figure 1b). Reaction retardation was greater in NaClO_4_ electrolytes, in which ∼60% removal was obtained within 20 min. In NaCl, however, the differences in degradation kinetics were less pronounced, with complete removal achieved after 5 min of electrolysis, which is consistent with oxidation of CBZ by the chlorine radical species. For both NAT/AT and BDD anodes in NaClO_4_ and NaCl electrolyte solutions, there was no discernible impact of the sulfate radical anion, SO_4_^·–^, on the observed degradation kinetics. The reaction rate constant between SO_4_^·–^ and CBZ is smaller than that between ^•^OH and CBZ but on the same order of magnitude (*k*_SO_4_^·–^,CBZ_ = 1.9 × 10^9^ M^–1^ s^–1^ vs *k*_^•^OH,CBZ_ = 8.8 × 10^9^ M^–1^ s^–1^).^7^ However, due to the low concentration of SO_4_^2–^ and thus SO_4_^·–^ present, CBZ degradation was dominated by O_3_, chlorine radical species, and ^•^OH.

#### FCZ

Very different degradation rates were obtained using the NAT/AT anode in NaClO_4_ vs NaCl electrolytes (Figure 1c). The rate constant between FCZ and O_3_ is 5 orders of magnitude lower than that of CBZ. However, faster-than-expected degradation was again observed with NAT/AT in NaClO_4_ electrolytes. 100% removal was attained in 5 min, which is notably faster than the predicted degradation rates considering reactions with both O_3_ and ^•^OH (Figure S2a). This result suggests that, similar to ibuprofen,^23^ FCZ degradation is promoted by anodic O_3_ activation on the NAT/AT anode. In NaCl solutions, on the other hand, FCZ removal was retarded compared to that in NaClO_4_ given that only 60–70% removal was observed after 1 h of electrolysis. This removal rate did not deviate significantly from our kinetic model predictions (Figure S2b), indicating that anodic O_3_ activation had little impact in this case.

Electrolysis using the BDD anode resulted in ∼80% FCZ removal in 20 min in NaClO_4_ and 80–90% removal in 1 h in NaCl electrolytes (Figure 1d). Higher removal efficiencies for BDD than NAT/AT in NaCl electrolytes are consistent with higher ^•^OH production levels at BDD coupled with an added contribution from direct electron transfer (*vide infra*). For FCZ, degradation with both electrodes was enhanced in the presence of SO_4_^2–^ in NaCl electrolytes. We propose that such enhancement in removal kinetics results from oxidation by sulfate radicals (SO_4_^·–^), which have a comparable or slightly higher redox potential (*E*^0^ = 2.5–3.1 V_NHE_)^31^ than that of ^•^OH (*E*^0^ = 2.7 V_NHE_).^32^ Formation of SO_4_^·–^ occurs via the following two pathways^32,33^
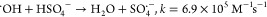
1
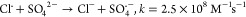
2with the second reaction, eq 2, occurring only when Cl^•^ (*E*^0^ = 2.4 V_NHE_)^34^ forms from Cl^–^ in solution. Even though [HSO_4_^–^] (p*K*_a_ = 1.92)^35^ is negligible in the bulk solution at pH ∼9, sulfate radical production via eq 1 could actually take place within the acidic (pH <2) electrical double-layer very close to the anode surface. Compared to ^•^OH, SO_4_^·–^ is a more selective oxidant that reacts primarily via electron transfer.^7,31,36^ While ^•^OH also reacts readily and rapidly via addition and H-abstraction pathways, SO_4_^·–^ generally has lower rate constants for those reactions. For many pharmaceutical compounds, however, reaction rates with SO_4_^·–^ are comparable to or occasionally faster than those with ^·^OH.^7^ In addition to FCZ, the presence of SO_4_^2–^ in wastewater matrices has been reported to accelerate the removal of other pharmaceutical compounds including ciprofloxacin, sulfamethoxazole,^22^ and ketoprofen^21^ using BDD anodes. With FCZ, the effect of SO_4_^2–^ was prominent in NaCl electrolytes. This result suggests that SO_4_^·–^ played a more important role in cases where O_3_ and/or ^·^OH-mediated oxidation was less important. In NaClO_4_ electrolytes, in contrast, FCZ removal appeared to be unaffected in the presence of SO_4_^2–^.

The higher removal percentage of FCZ than CBZ at BDD over 20 min even though the reaction rate constant between FCZ and ^·^OH is slightly lower (*k*_FCZ,^·^OH_ = 4.4 × 10^9^ M^–1^ s^–1^ vs *k*_CBZ,^·^OH_ = 8.8 × 10^9^ M^–1^ s^–1^)^8,26^ suggests that additional oxidation pathways are operative. Given that direct electron transfer (DET) is known to happen at the surface of BDD (but not NAT/AT), it was suspected that DET made a substantial contribution to the removal of FCZ. DET from FCZ to BDD was confirmed using linear sweep voltammetry (LSV) in 50 mM NaClO_4_ (control) and in 50 mM NaClO_4_ containing 20 μM CBZ or FCZ (Figure S3). While no obvious feature is observed for CBZ compared to the NaClO_4_ control, a notable peak was recorded at ∼3.0 V_NHE_ in the FCZ voltammogram, which implies that DET is taking place.

**Figure 2:**
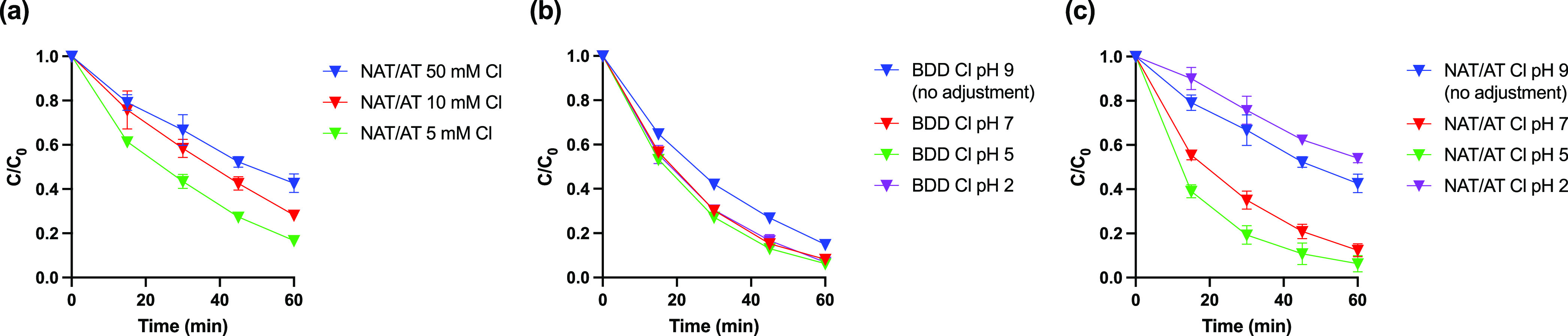
(a) FCZ degradation by NAT/AT under different chloride concentrations (50, 10, and 5 mM NaCl, 5 mM NaClO_4_ was added to 5 mM NaCl to compensate for lower conductivity). Influence of pH on FCZ degradation by (b) BDD and (c) NAT/AT in 50 mM NaCl electrolytes. For degradation under pH 9 (no adjustment), no phosphate buffer was added.

The FCZ degradation results on both anodes in NaCl electrolytes indicate that FCZ is relatively resistant to oxidation by both free chlorine and chlorine radical species. This hypothesis is supported by a separate control experiment using a NaOCl solution (<30% removal in 2 h with excess NaOCl, data not shown) as well as experiments at NAT/AT with variable Cl^–^ concentrations. When [Cl^–^] is decreased from 50 to 5 mM, removal of FCZ after 1 h increased from 60 to >80% (Figure 2a), which results from higher O_3_ production with lower [Cl^–^].^23^

The influence of pH on FCZ degradation was also investigated using both electrolytes with pH adjusted to 5, 7, and 9 in phosphate buffers. In some cases, pH 2 was also examined for trend elucidation. In NaClO_4_, pH had negligible influence on FCZ removal for NAT/AT and BDD (Figure S4). Similar degradation kinetics at all pH values indicate a consistent ·OH production on the anode surface coupled with O_3_ production on NAT/AT. In NaCl, different kinetic profiles for BDD compared to NAT/AT were observed. For example, degradation on BDD anode was not influenced by the variation in electrolyte pH in that similar removal levels were observed as pH was lowered from 9 to 2 (Figure 2b). This result, along with the similar result in Figure S4, also suggested that phosphate buffer did not influence degradation kinetics. On the NAT/AT anode, on the other hand, faster FCZ degradation was recorded as pH was lowered from 9 to 5 (e.g., ∼60% degradation at pH 9 to >90% degradation at pH 5 after 1 h of electrolysis). However, the trend did not continue to pH 2, where the slowest removal was observed (Figure 2c).

To explain the differences in FCZ degradation vs pH, chlorine evolution was measured for each anode, and O_3_ evolution was measured only on the NAT/AT anode. Chlorine evolution, due to the production of reactive chlorine species, intrinsically leads to an increase in solution pH because of the depletion of H^+^ at cathode comparing to OH^–^ at anode. During the chlorine evolution experiments, pH quickly rose to ∼9 from circumneutral pH. On the other hand, it is also known that solution pH affects chlorine evolution kinetics.^37^ With BDD, stable chlorine evolution rates as well as current efficiencies were recorded at all pH values from 2 to 9 (Figure S5a). With NAT/AT, on the other hand, decreased chlorine evolution (CER) and lower current efficiencies were observed as the pH was decreased (Figure S5b). Meanwhile, the aqueous O_3_ concentrations followed an opposite trend in that during 15 min of electrolysis, the steady state [O_3_] increased from ∼0.5 mg/L at pH 9 to ∼2 mg/L at pH 2 (Figure S6). These results suggest that, at NAT/AT in the presence of Cl^–^, lower pH values favor the production of O_3_ over chlorine evolution, which can explain the FCZ degradation trend in the pH range from 9 to 5. At pH 2, despite higher O_3_ production, the slowest FCZ removal can be attributed to the different reactivities between deprotonated and protonated species with O_3_.^38^ In many cases, the reaction rate of the deprotonated species of a compound can be several orders of magnitude higher than that of its protonated species. FCZ has p*K*_a_ values of 2.6, 2.9, and 11.0, where 2.6 and 2.9 correspond to the two nitrogens in the triazole rings.^39^ At pH 2, a protonated nitrogen could have significantly lower or no reactivity with O_3_, resulting in retarded removal. Though kinetic data is not available for FCZ, the deprotonated form of imidazole, with a similar structure to triazole, reacts with O_3_ 4 orders of magnitude faster compared to its protonated form.^38^

### Transformation Product Formation, Identification, and Removal

**Figure 3:**
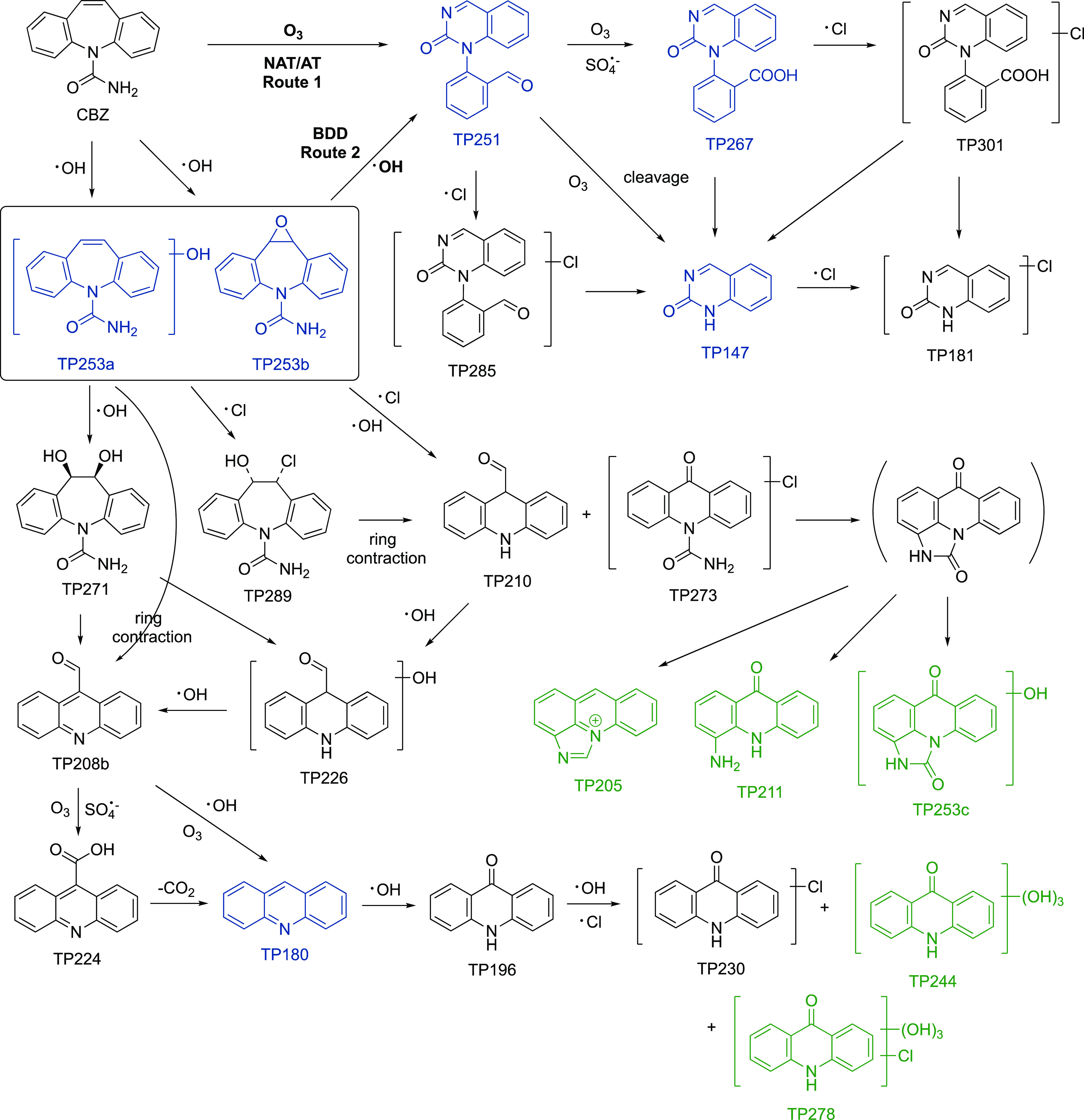
Proposed carbamazepine (CBZ) transformation pathways. Blue structures mark products detected during electrolysis in NaClO_4_ electrolytes. Green structures mark products with higher confidence levels/higher uncertainties and thus more tentative structures and pathways.

Liquid chromatography–mass spectrometry (LC-MS) analysis showed that there were 23 distinct transformation products (TPs) observed during CBZ oxidation, 10 of which have not been previously reported. Properties of the TPs as documented in Table 1 include the corresponding retention times (RT), the measured *m*/*z* ratios, major fragment ions, mass error to theoretical *m*/*z* ratios, calculated chemical formulas, and proposed structures and their confidence levels. Reaction pathways for TP formation are proposed in Figure 3 based on this information and further TP analysis (*vide infra*). The pathways presented, as will be shown later, may not be comprehensive, and formation of many TPs is possible via multiple pathways.

**Table 1:**
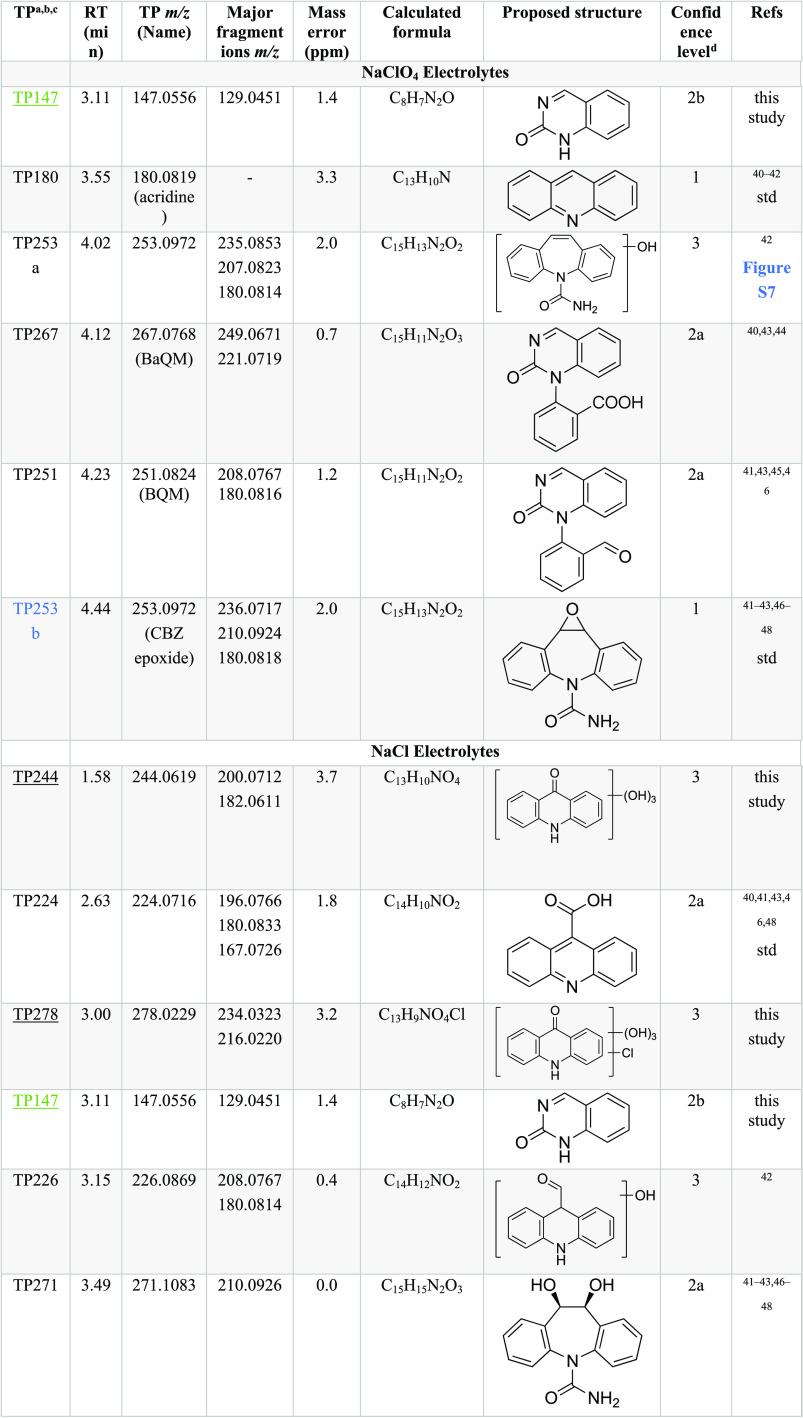

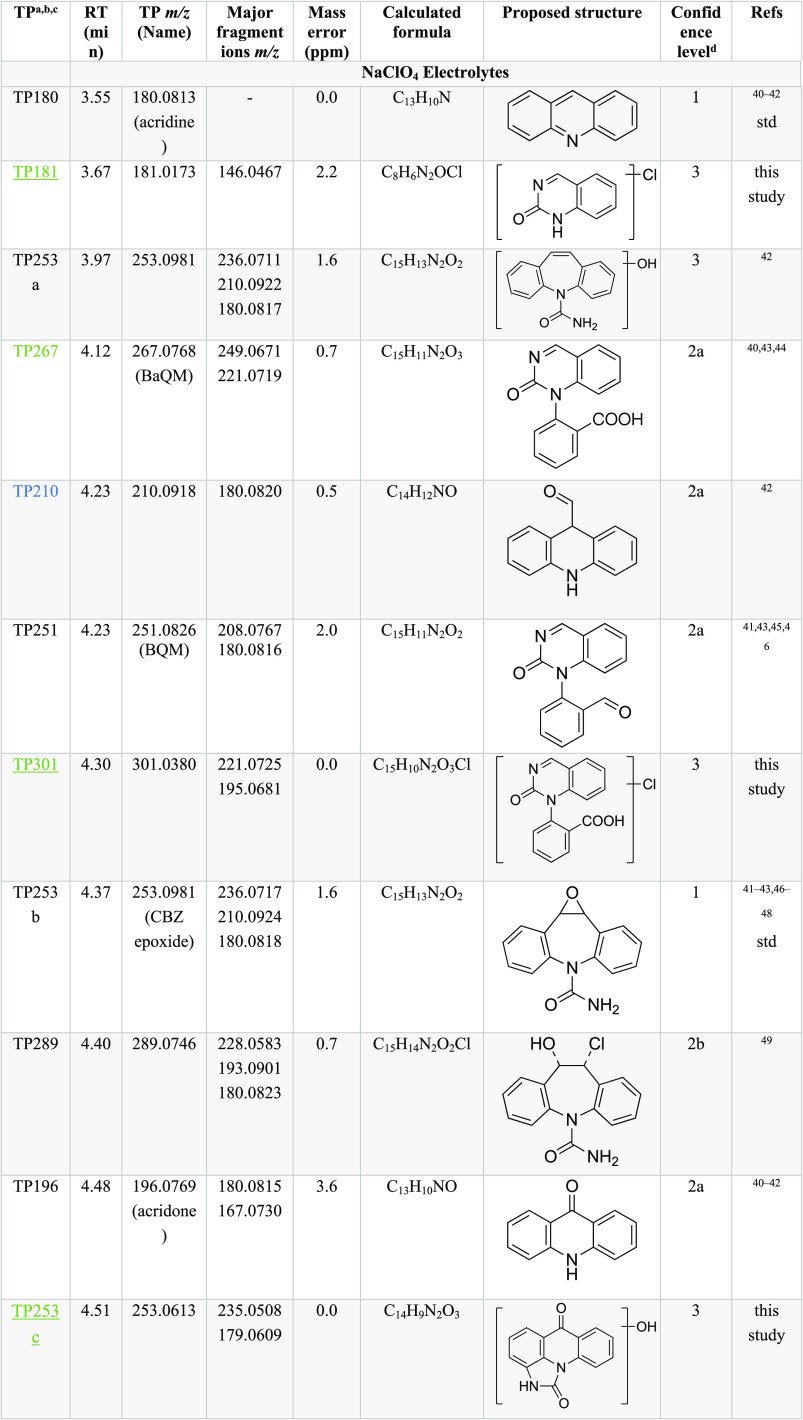

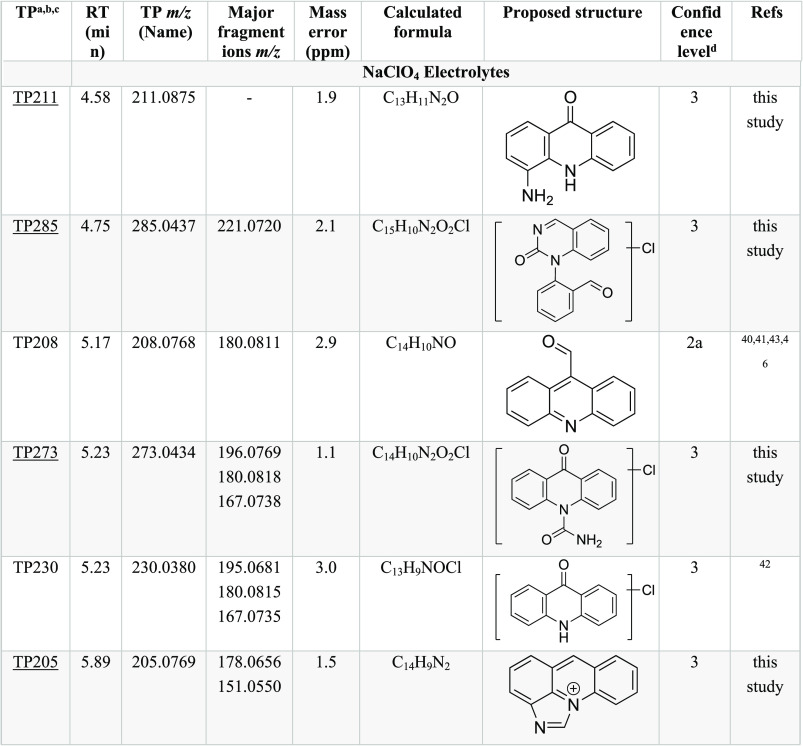
Carbamazepine (CBZ) Transformation Products^a^^b^^c^^d^

a TPs marked with underline have, to the best of our knowledge, not been reported before.

b TPs marked in green are not detected at BDD, and TPs marked in blue are not detected at NAT/AT, in respective electrolytes.

c Mass spectra of TPs containing Cl in their chemical formula are provided in Figure S8.

d Confidence levels are assigned based on the level system proposed by Schymanski et al.^50^ for emerging pollutant transformation product identification. The meaning of each level is: level 1, confirmed structure by reference standard; level 2, probable structure by 2a, literature matching of fragmentation patterns and 2b, diagnostic evidence where only one structure fits the experimental information; level 3, tentative candidate where either the structure is tentative and/or substitution positions are uncertain. For level 3 TPs where structure rather than substitution position is uncertain, tentative structure is proposed based on fragmentation analysis when available (Table S2).

#### CBZ in NaClO_4_ Electrolytes

**Figure 4:**
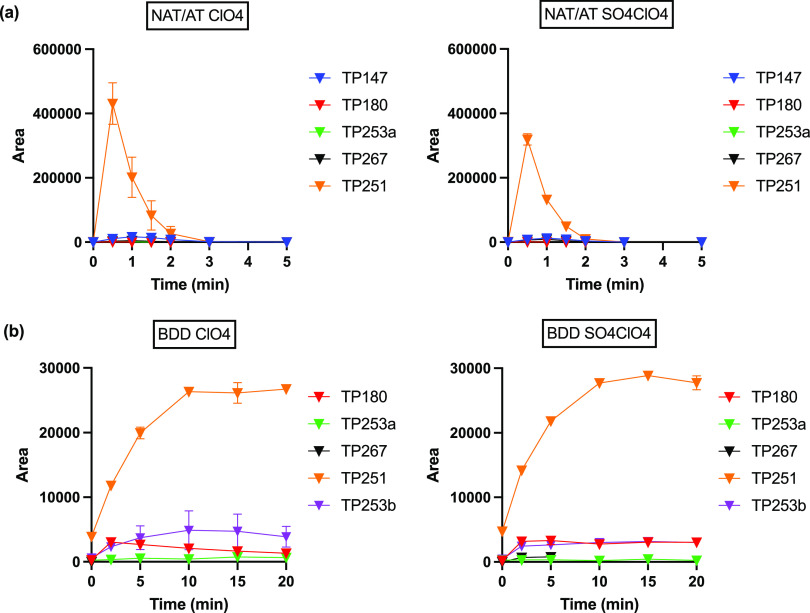
CBZ transformation product evolution in NaClO_4_ electrolytes at (a) NAT/AT and (b) BDD.

In NaClO_4_ electrolytes, 5 TPs (TP147, TP180, TP253a, TP267, and TP251) were detected using NAT/AT and 5 (TP180, TP253a, TP267, TP251, and TP253b) using BDD (Figure 3, labeled blue). For NAT/AT, TP251 (*m*/*z* = 251.0824, C_15_H_11_N_2_O_2_) showed an MS response 1 order of magnitude higher than all of the other TPs in NaClO_4_-based electrolytes (Figure 4a). This TP was identified as 1-(2-benzaldehyde)-4-hydro-(1H,3H)-quinazoline-2-one (BQM), which has been previously reported as a major CBZ ozonation product (Figure 3, route 1).^43,45^ Another identified product was TP267 (*m*/*z* = 267.0768, C_15_H_11_N_2_O_3_). While various structures have been proposed for the same *m*/*z* ratios in the literature, based on its fragmentation patterns, TP267 was identified as the carboxylic acid of TP251 (BaQM). The oxidation of BQM (TP251) to BaQM (TP267) has been reported before during ozonation.^43^ Other pathways for BaQM formation were also seen during biological^40^ and ferrate^44^ oxidation of CBZ, where BQM was not detected. Similar to the oxidation of BQM to BaQM, an aldehyde-to-carboxylic-acid pathway has been reported in a variety of treatment options including ozonation,^45^ UV/chlorine,^42^ UV/TiO_2_ photocatalysis,^47^ ferrate,^44^ and biodegradation with white-rot fungus *Pleurotus ostreatus*.^41,46^ In our case, the abundance of TP267 increased significantly when SO_4_^2–^ was added in the electrolyte solution (Figure S9a), which implies that SO_4_^·–^ is also capable of oxidizing aldehydes to carboxylic acids.^48,49^ Following TP251 and TP267, TP147 (*m*/*z* = 147.0556, C_8_H_7_N_2_O) was formed via bond cleavage. Due to the fast reaction between CBZ and O_3_, oxidation by ^·^OH played a minor role in oxidation with NAT/AT. The hydroxylated product, TP253a (*m*/*z* = 253.0972, C_15_H_13_N_2_O_2_), was detected with the lowest response among all 5 TPs, with its maximum peak area 3 orders of magnitude less than that of TP251.

For BDD electro-oxidation, the TP with the highest response in the MS was also TP251, although the maximum peak area was 1 order of magnitude smaller compared to that for NAT/AT (Figure 4b), which can be explained by the longer treatment time. Another TP with *m*/*z* = 253.0972 (TP253b) was detected, which was not seen during NAT/AT electrolysis. This product is carbamazepine 10,11-epoxide (CBZ-EP) based on direct comparison to commercial standard. The peak area of CBZ-EP stayed relatively constant during 20 min of electrolysis (Figure S9b). In the case of BDD-induced ^·^OH-mediated oxidation, in comparison to the direct formation of TP251 (BQM) via ozonation, we propose that CBZ is first oxidized to its hydroxylated product (TP253a), which is in turn oxidized to BQM (Figure 3, route 2). The bond cleavage product TP147 was not detected during BDD electrolysis in NaClO_4_ electrolytes. Additionally, TP267 was detected at very low levels only in the presence of SO_4_^2–^, which indicates that ·OH was less effective than SO_4_^·–^ for the oxidation of aldehydes to carboxylic acids.

Finally, electrolysis in NaClO_4_ led to the formation of TP180 (*m*/*z* = 180.0819, C_13_H_10_N), which was confirmed using a reference standard to be acridine, a known mutagenic and carcinogenic compound.^11^ TP180 is relatively stable. It forms in CBZ stock solutions and, in cases where the stock was kept for longer times, was actually detected without electrolysis. TP180 is also transformed slowly by ^·^OH. Comparing to the relatively fast removal by O_3_ with NAT/AT, it was only slowly removed using BDD (Figure S9b).

Removal of the TPs was slower in comparison to parent compound degradation. The 5 TPs detected using NAT/AT were formed and basically completely removed within 5 min of electrolysis (Figures 4a and S9a). For BDD, the TP concentrations leveled off after 20 min, after which their concentrations started to decrease due to further oxidation (Figures 4b and S9b). This result indicates that longer treatment times are required for further oxidative removal of the TPs where ^·^OH is the sole oxidant.

#### CBZ in NaCl Electrolytes

**Figure 5:**
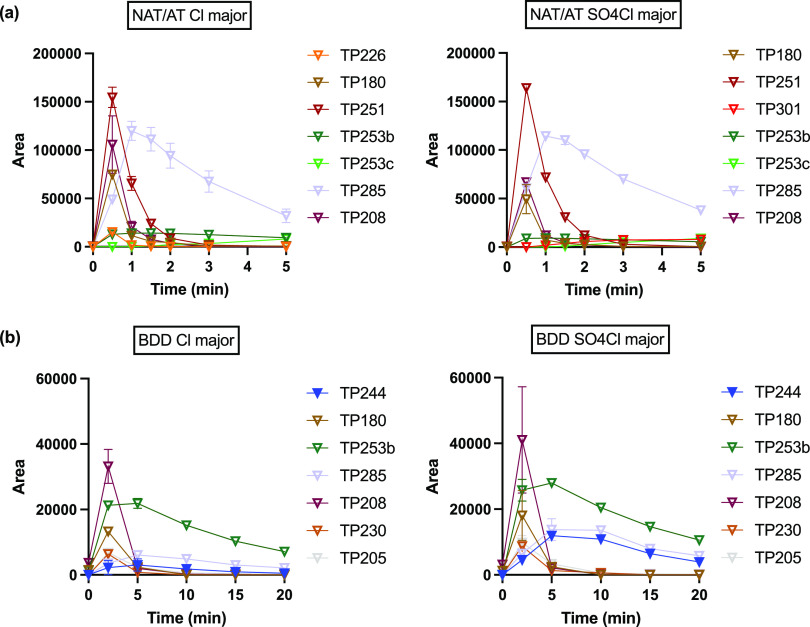
Major CBZ transformation product evolution in NaCl electrolytes at (a) NAT/AT and (b) BDD.

In NaCl electrolytes, more TPs were detected compared to those found during electrolysis in NaClO_4_. During electrolysis in NaCl, 22 and 18 out of all 23 TPs were detected using NAT/AT and BDD anodes, respectively. For NAT/AT, TP251 was again the highest response TP with maximum peak intensity occurring at 0.5 min (Figure 5a). In this case, unlike that in NaClO_4_, it was no longer the overwhelming peak in the MS. If we assign the peak area of TP251 at 0.5 min to be a response of 1.0, then 7 of the other TPs had maximum responses of >0.05. TP285 (*m*/*z* = 285.0437, C_15_H_10_N_2_O_2_Cl) had a response of ∼0.74 and was identified as the chlorination byproduct of TP251. TP253b (CBZ-EP), the epoxide product mediated by ^·^OH oxidation, was also detected at substantial levels in NaCl compared to that in NaClO_4_. Another set of interesting TPs included TP208 (*m*/*z* = 208.0768, C_14_H_10_NO), TP224 (*m*/*z* = 224.0716, C_14_H_10_NO_2_), TP180, and TP196 (*m/z* = 196.0768, C_13_H_10_NO). TP208 was determined to be 9-acridine-carboxaldehyde. Possible precursors for it include TP253a and TP253b (*vide infra*). The pathway TP253b → TP208 has been reported during ozonation,^43^ ClO_2_ oxidation,^51^ and biotransformation^46,51^ by ring contraction and loss of the carbamoyl group. Oxidation of the aldehyde group on TP208 gives the corresponding carboxylic acid product TP224 (9-acridinecarboxylic acid), which can undergo decarboxylation to give TP180, acridine. TP208 and TP224 represent another aldehyde–carboxylic acid pair like TP251 and TP267. Hydroxylation and oxidation of TP180 then leads to the formation of TP196, acridone, which also had a relative response factor of >0.05. The oxidation of acridine to acridone has been confirmed in electrochemical oxidation^39^ and represents a known biological detoxification process.^46^ The sequence of pathways from TP208 to TP196 has been observed in biotransformation in WWTP biological processes^40^ as well as with the white-rot fungus *P. ostreatus*.^41,46^ Direct formation of TP180 from TP208 by cleavage of the aldehyde group is also possible.^46,51^ In addition to the above 4 TPs, TP226 (*m*/*z* = 226.0869, C_14_H_12_NO_2_) could be another hydroxylated precursor of TP208, and TP253c (*m*/*z* = 253.0613, C_14_H_9_N_2_O_3_) is proposed to form via an intramolecular cyclization at the carbamoyl group. Overall, since O_3_ production is inhibited in the presence of Cl^–^, other oxidation pathways played more important roles in the formation of the other TPs.

Many additional TPs were observed using NAT/AT although with lower relative responses. In addition to TP285, which is the chlorinated byproduct of TP251, TP301 (*m*/*z* = 301.0380, C_15_H_10_N_2_O_3_Cl), TP181 (*m*/*z* = 181.0173, C_8_H_6_N_2_OCl), TP289 (*m*/*z* = 289.0746, C_15_H_14_N_2_O_2_Cl),^49^ and TP230 (*m*/*z* = 230.0380, C_13_H_9_NOCl) were identified as the chlorinated byproducts of TP267, TP147, TP253b, and TP196, respectively. TP271 (*m*/*z* = 271.1088, C_15_H_15_N_2_O_3_) was identified as 10,11-dihydro-10,11-*cis*-dihydroxy-carbamazepine (*cis*-diOH-CBZ). TP205 (*m*/*z* = 205.0769, C_14_H_9_N_2_) and TP211 (*m*/*z* = 211.0875, C_13_H_11_N_2_O) were two other products, besides TP253c, that may have formed after carbamoyl group intramolecular cyclization. This cyclization pathway has also been proposed in the treatment of CBZ by UV/chlorine and UV/H_2_O_2_.^42,48,52^ Finally, TP230 (*m*/*z* = 230.0380, C_13_H_9_NOCl), TP244 (*m*/*z* = 244.0619, C_13_H_10_NO_4_), and TP278 (*m*/*z* = 278.0229, C_13_H_9_NO_3_Cl) are proposed to be the singly and multiply hydroxylated and chlorinated product of TP196, acridone.

Formation patterns of several TPs were different when SO_4_^2–^ was present in the electrolyte. Like TPs formed in NaClO_4_ electrolytes, the relative response factor of TP267 was significantly higher (Figure S10a) along with its corresponding chlorinated byproduct, TP301 (Figure S10b). Another carboxylic acid product, TP224, was also detected in higher abundance when SO_4_^2–^ was added to the base electrolyte despite TP208 having a lower response (Figure S10c). Meanwhile, lower response factors for several other TPs were observed. The more prominent ones include TP226, TP271, and TP253a (Figure S10d,e). These collective observations indicate that, in the presence of SO_4_^2–^, oxidation was primarily promoted by ^·^OH and SO_4_^·–^ simultaneously along with subsequent chlorination. However, ^·^OH played a smaller role in oxidation comparing to the cases in which SO_4_^2–^ was absent. Since electron transfer is the preferred oxidation pathway by SO_4_^·–^ as opposed to addition or H-abstraction, the hydroxylated products produced by ^·^OH oxidation were formed in lower yields.

In the case of BDD electrolysis, the product with the highest response was TP208 with an intensity that peaked at 0.5 min (Figure 5b). TP208 was followed by TP253b and TP180 with relative response factors of ∼0.59 and 0.36, respectively. Like the reactions occurring in NaClO_4_ electrolytes, the maximum MS peak area was also 1 order of magnitude smaller than that for NAT/AT. TP147 and TP267, as well as their chlorinated byproducts TP181 and TP301, were not detected during BDD electrolysis at measurable levels in both solutions. Furthermore, TP251 appeared to be a less important TP, which suggests that other oxidation pathways predominated over route 2 in Figure 3. Overall, the same mixture of TPs was observed using BDD as those found with NAT/AT.

CBZ was completely degraded within 1 min of electrolysis using NAT/AT. Most of the 22 TPs detected during NAT/AT electrolysis peaked between 0.5 and 1 min and then were either removed completely or to a high degree after 5 min (Figures 5a and S11a). For BDD, 100% CBZ removal occurred around 5 min, whereas most of the TPs peaked between 2 and 5 min and were then partially removed after 20 min of electrolysis (Figures 5b and S11b). These results indicate the effectiveness of O_3_ in eliminating the intermediate TPs in addition to the initial oxidation step of CBZ. Responses of all TPs in MS at the two electrodes in all four electrolyte combinations are summarized in Table S3.

#### CBZ TP Quantification and Pathway Elucidation

The more important TPs of CBZ were confirmed and quantified where commercial standards are available: TP180 (acridine), TP253b (carbamazepine 10,11-epoxide), and TP224 (9-acridinecarboxylic acid). TP180 and TP224 are also known to be toxic. More of all of the three TPs was detected in NaCl than in NaClO_4_ electrolytes. Peak concentrations of ∼1.8, 2.0, and 0.054 μM were recorded for TP180, TP253b, and TP224, respectively (Figure S12). In all electrolytes and in secondary effluent (discussed below), these 3 TPs did not constitute a major part (<15%) of the transformed CBZ in terms of mass balance. Mass balance is not closed here due to the variety of TPs detected and a lack of reference standards. However, identification over complete quantification of the TPs and of their removal trends can be more important for practical engineering purposes, since TP distributions can vary a lot depending on the wastewater composition, yet TP identification can help facilitate treatment efficiency evaluation and toxicity assessment in general.

Electrolysis using the TP253b and TP224 as parent compounds was also conducted to further elucidate and confirm the transformation pathways in Figure 3 (more details in Figures S13 and S14). In general, the formation of many TPs is possible via multiple pathways.

#### FCZ

**Figure 6:**
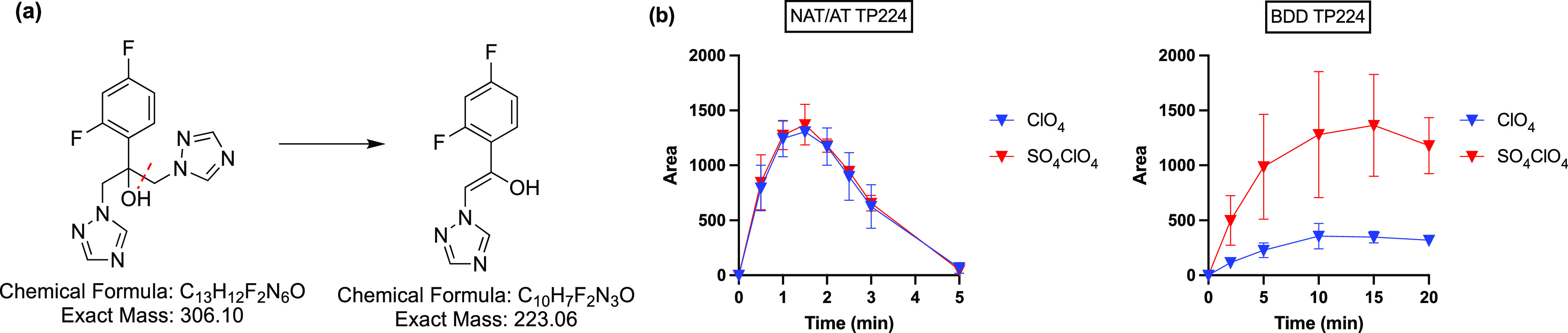
(a) Proposed formation pathway and (b) evolution of FCZ transformation product TP224 at NAT/AT and BDD.

A lot less TPs (<10 total) have been reported for FCZ in the literature compared to those for CBZ,^12,25,53−56^ ranging from 0 detected in constructed wetlands^56^ to 6 under UV/chlorine.^12^ In NAT/AT and BDD systems, only 1 TP, TP224 (*m*/*z* = 224.0643, C_10_H_8_F_2_N_3_O), was detected in the MS for FCZ degradation in NaClO_4_ electrolytes. It formed from cleavage of the parent molecule (Figure 6a). This product has been reported previously as a degradation product during treatment with UV/chlorine and with H_2_O_2_ solutions.^12,53^ TP224 is predicted to have a higher toxicity than FCZ.^12^ During NAT/AT electrolysis, TP224 peaked at 1.5 min and was completely removed after 5 min, while with BDD, its concentration leveled off at ∼20 min (Figure 6b). The absence of TP224 in NaCl electrolytes suggests that it could be susceptible to attack by chlorine radical species. Given the lack of detectable TPs, it appears that a fast total oxidation of the intermediate products takes place compared to the more persistent parent molecule FCZ.

### Pharmaceutical Removal in Environmental Waters

The oxidative degradation of CBZ and FCZ and formation of their transformation products were also carried out in actual latrine wastewater and in secondary effluent along with a mixture of common pharmaceutical compounds. Previously, Lee et al. characterized micropollutant elimination during ozonation into five categories based on their reaction rate constants with O_3_ and ^·^OH.^6,26^ CBZ and FCZ fall into group Ia with the fastest kinetic rates and group IV with the slowest kinetic rates, respectively. To obtain a quantitative ranking of pharmaceutical degradation with NAT/AT, five other pharmaceuticals in the top 100 list^1^ were selected from the different categories. They included atenolol (ATL, group Ib), gabapentin (GBP, group II), trimethoprim (TMP, group Ia), sulfamethoxazole (SMX, group Ia), and ibuprofen (IBP, group III). The physical–chemical properties of the seven target compounds including their p*K*_a_ values and rate constants with O_3_ and ^·^OH are summarized in Table S4. Individual degradation kinetics of ATL, GBP, and IBP at NAT/AT and their kinetic modeling predictions are shown in Figure S15.

**Figure 7:**
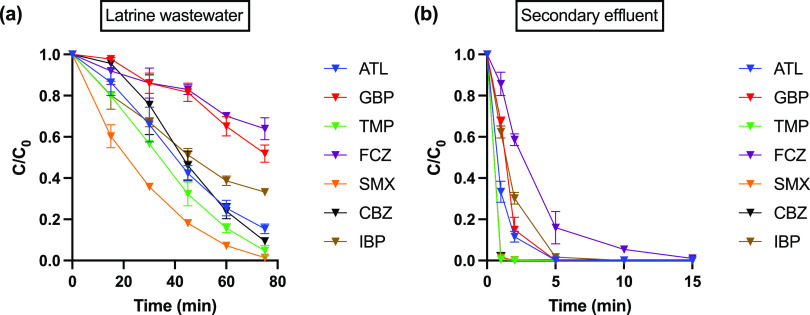
Removal of spiked pharmaceutical compounds (2 μM each) during electrolysis of (a) latrine wastewater and (b) secondary effluent by NAT/AT.

The 7 pharmaceuticals were spiked into the wastewaters at 2 μM each, which is higher than the typical concentrations detected in wastewater sources (ranging from 0 to several thousand ng/L), to investigate treatment with elevated concentrations.^1^ The chemical compositions of the wastewaters are given in Table S5. In latrine wastewater, due to the presence of high background levels of COD (440 mg/L) and NH_4_^+^ (31 mM), and the resulting competitive consumption of oxidants, degradation was retarded compared to that in pure electrolytes. In latrine wastewater, >80% removal of the spiked pharmaceutical compounds was achieved in 75 min electrolysis except for FCZ, GBP, and IBP (Figure 7a), indicating longer treatment times were required. In addition, a COD reduction of ∼300 mg/L was attained simultaneously (Figure S16). In domestic secondary wastewater treatment effluent, the pharmaceuticals were degraded faster in comparison to latrine wastewater due to low initial COD (80 mg/L) and NH_4_^+^ (0.3 mM) concentrations. Complete removal of all pharmaceuticals except for FCZ was achieved in 5 min of electrolysis, while the time required for FCZ removal was 15 min (Figure 7b), at which point the complete removal of COD was also obtained. 8 of the TPs of CBZ (TP147, TP271, TP180, TP253a, TP267, TP251, TP253b, and TP208) and TP224 of FCZ were detected. Accompanying parent compound degradation, complete removal of all 9 TPs for both CBZ and FCZ was also achieved in 15 min (Figure S17). Compared to NAT/AT, BDD, while achieving similar COD reduction (Figure S16), demonstrated better performance for pharmaceutical degradation in latrine wastewater and worse performance in secondary effluent (Figures S18 and S19). This result indicates the advantage of NAT/AT application in systems with lower chloride concentration. Potential improvement could also be achieved by including pretreatment units to lower COD and remove NH_4_^+^ and other interfering components from wastewaters before electrochemical treatment.

## Conclusions

In summary, the NAT/AT anodes demonstrated promising performance in degrading a range of pharmaceutical compounds (with very different reactivities with O_3_) as well as their transformation products. The NAT/AT-SS system, requiring lower cell voltage than BDD (Table S5) under the same applied current density, could achieve similar or better removal with lower energy consumption. It thus potentially represents a more economical and efficient method for water treatment practices that is capable of large-scale implementation. Moreover, the secondary effluent used herein had similar chemical compositions to a biologically treated hospital wastewater previously investigated,^22^ suggesting that electrolytic oxidation with NAT/AT could also provide a suitable treatment alternative for the control of pharmaceuticals in hospital wastewaters.

## Supporting Information Available

Experimental results and kinetic modeling predictions for pharmaceutical degradation; linear sweep voltammograms; chlorine evolution rate and current efficiency; dissolved O_3_ concentration measurements; mass spectra of the chlorinated transformation products; CBZ transformation product evolution; quantification of TP180, TP253b, and TP224; electrochemical oxidation and transformation product evolution of TP253b and TP224; COD removal during treatment of latrine wastewater spiked with pharmaceuticals; kinetic modeling description; principle reactions in the kinetic model; target pharmaceutical compound properties; composition of latrine wastewater and secondary effluent (PDF)
