# Supplementary material for: Ozone- and Hydroxyl Radical-Mediated Oxidation of Pharmaceutical Compounds Using Ni-Doped Sb–SnO2 Anodes: Degradation Kinetics and Transformation Products
Source: ACS ES T Eng. 2023 Jan 26;3(3):335–48. doi: 10.1021/acsestengg.2c00337 (PMC10012175; doi:10.1021/acsestengg.2c00337)
Supplement: Supplementary file 1 — ee2c00337_si_001.pdf [file ee2c00337_si_001.pdf]

Supporting Information

Ozone- and hydroxyl radical-mediated oxidation of pharmaceutical compounds using Ni-doped Sb-SnO_2 anodes: Degradation kinetics and transformation products

Yi Zhang[1]*, Lei Guo[1,2], and Michael R. Hoffmann[1]

1. Linde Laboratories, California Institute of Technology, Pasadena, California 91125, United States
2. Department of Civil Engineering, University of Arkansas, Fayetteville, Fayetteville, Arkansas 72701, United States

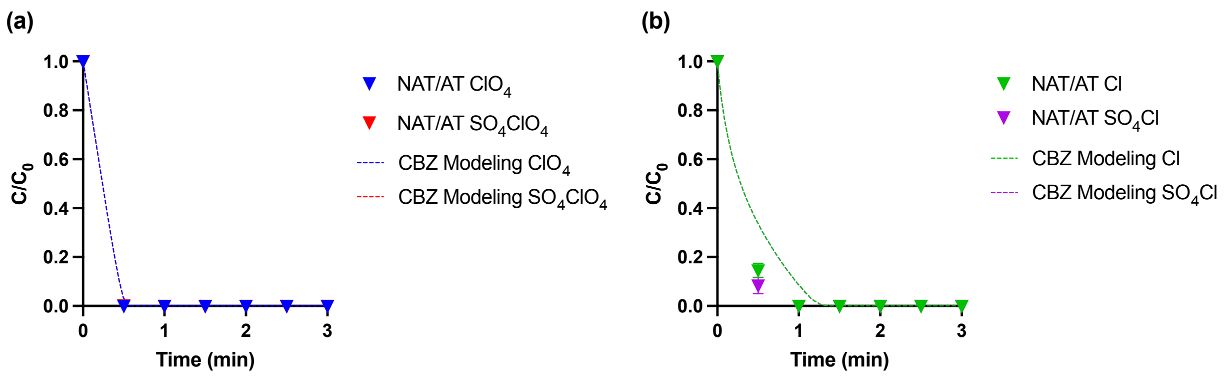

Figure S1. Experimental results and kinetic modeling predictions (dashed lines) for CBZ degradation by NAT/AT in (a) ClO_4: 50 mM $NaClO_4$ and SO_4ClO_4: 5 mM Na_2SO_4 + 50 mM $NaClO_4$ and (b) Cl: 50 mM NaCl and SO_4Cl: 5 mM Na_2SO_4 + 50 mM NaCl electrolytes.

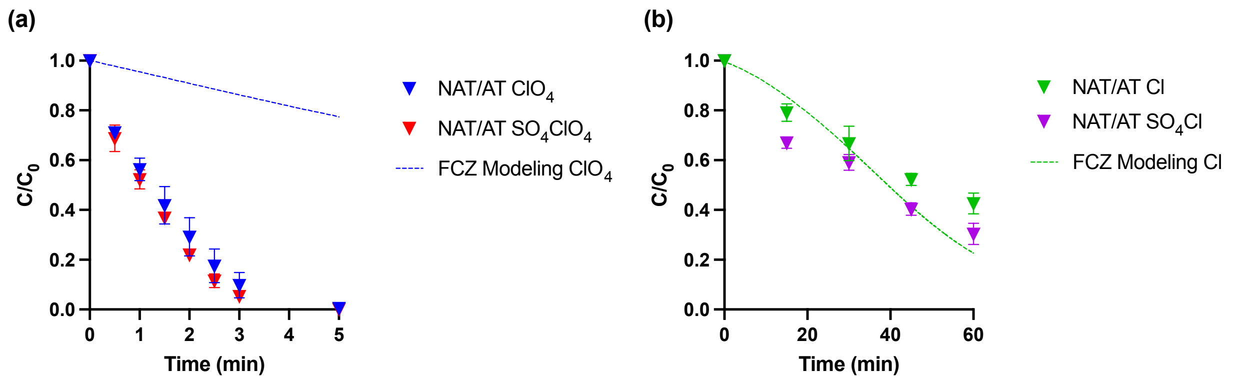

Figure S2. Experimental results and kinetic modeling predictions (dashed lines) for FCZ degradation by NAT/AT in (a) ClO_4: 50 mM $NaClO_4$ and SO_4ClO_4: 5 mM Na_2SO_4 + 50 mM $NaClO_4$ and (b) Cl: 50 mM NaCl and SO_4Cl: 5 mM Na_2SO_4 + 50 mM NaCl electrolytes.

Modeling result in SO_4Cl (5 mM Na_2SO_4 + 50 mM NaCl) did not reflect the faster FCZ degradation in the presence of SO_4^{2-} as recorded in the experiments, suggesting that other pathways may potentially produce $SO_4^{\cdot -}$ in addition to the homogeneous reactions (reaction 60 through 78 in Table S1). One possibility is that $SO_4^{\cdot -}$ was generated electrochemically like ·OH,

O_3, and chlorine radicals. Taking an estimated zero-order rate constant of ~1×10^{-8} s^{-1} for $SO_4^{\cdot -}$ production, the model was able to reproduce a similar enhanced degradation kinetics.

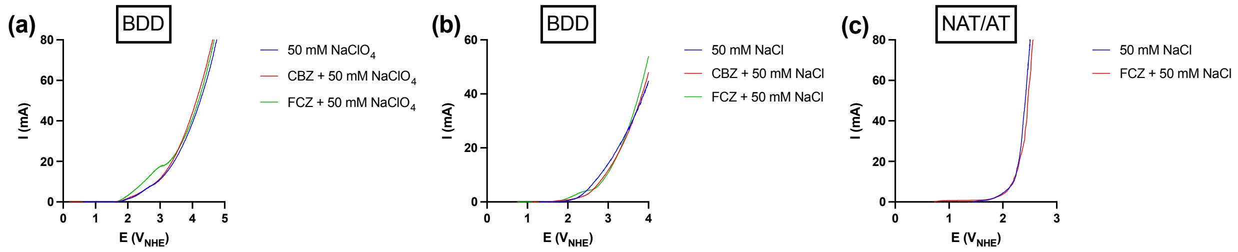

Figure S3. Linear sweep voltammograms (LSV) of (a) BDD in 50 mM $NaClO_4$, (b) BDD in 50 mM NaCl, and (c) NAT/AT in 50 mM NaCl electrolytes in the absence and presence of 20 μM CBZ or FCZ.

Evidence for direct electron transfer (DET) at BDD is suggested given the peak around 3.0 V_{NHE} in the FCZ + 50 mM $NaClO_4$ voltammogram, which was absent in the 50 mM $NaClO_4$ control voltammogram. A similar but less pronounced peak around 2.5 V_{NHE} in the FCZ + 50 mM NaCl voltammogram suggested that DET also happens in the presence of chloride but probably with less intensity. In both cases, no additional features were observed for CBZ compared to the 50 mM $NaClO_4$ or NaCl control voltammograms. On the other hand, DET is not known to happen at NAT/AT, which is also confirmed by the absent of DET peaks in the NAT/AT voltammograms.

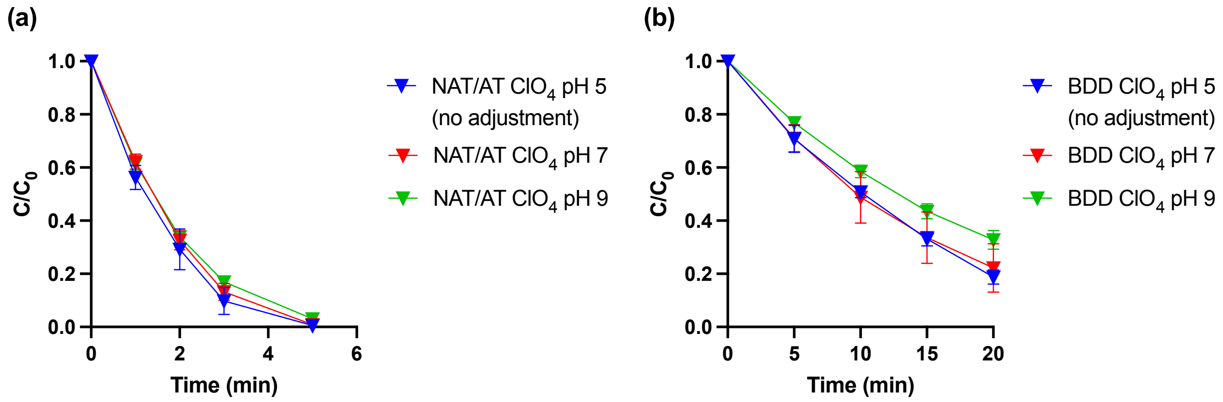

Figure S4. FCZ degradation by (a) NAT/AT and (b) BDD in 50 mM $NaClO_4$ electrolytes with pH adjusted to 5, 7, and 9 in phosphate buffers. For degradation under pH 5 (no adjustment), no phosphate buffer was added.

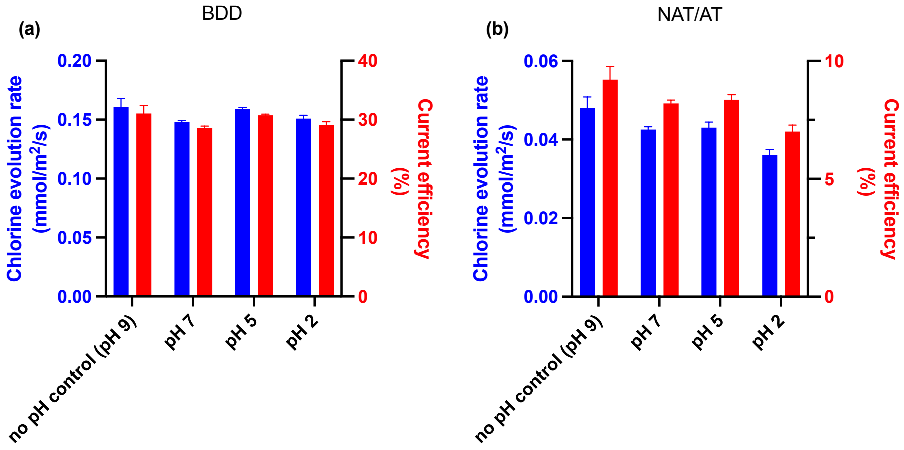

Figure S5. Chlorine evolution rate and current efficiency measured in 50 mM NaCl electrolytes during electrolysis by (a) BDD and (b) NAT/AT.

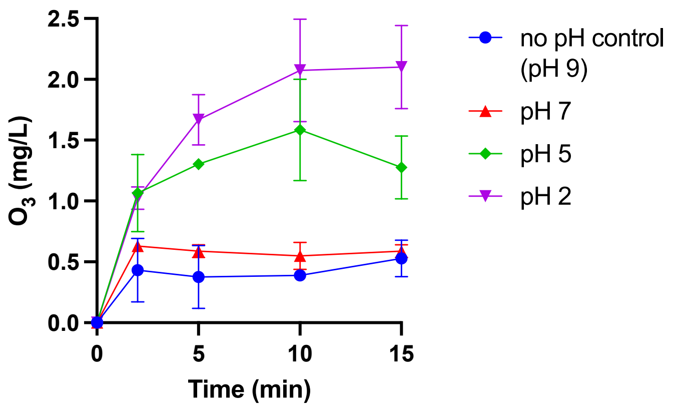

Figure S6. Dissolved O_3 concentrations measured under different pHs in 50 mM NaCl electrolytes during electrolysis by NAT/AT.

The kinetic model showed that reaction between O_3 and OCl^- ($k_{O_3,OCl^-} = 120\ M^{-1}s^{-1}$ *vs.* $k_{O_3,HOCl} < 0.002\ M^{-1}s^{-1}$) did not play an important role in affecting $[O_3]$ under higher pH. At pH 9, reaction between O_3 and OH^- led to slight decrease in aqueous $[O_3]$. Most of the observed difference in $[O_3]$, however, should come from different O_3 production at NAT/AT.

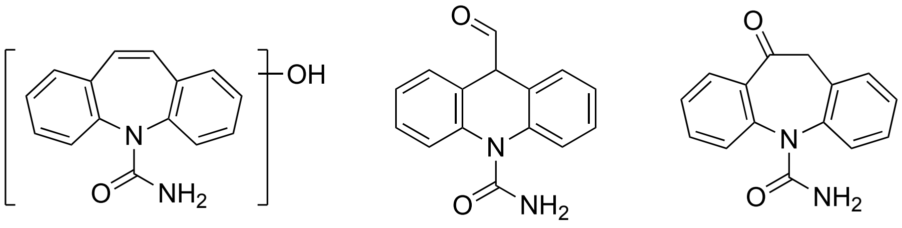

Figure S7. Possible structures for TP253a.

Several structures for this hydroxy-CBZ TP have been proposed in literature. The structures shown have been proposed during ozonation,[1] heterogeneou photocatalysis,[2] UV/chlorine,[3,4] chlorination,[5] UV/H_2O_2,[6] photodegradation,[7] and $UV/S_2O_8^{2-}$.[8] However, since a standard is not available, its exact structure cannot be determined.

80

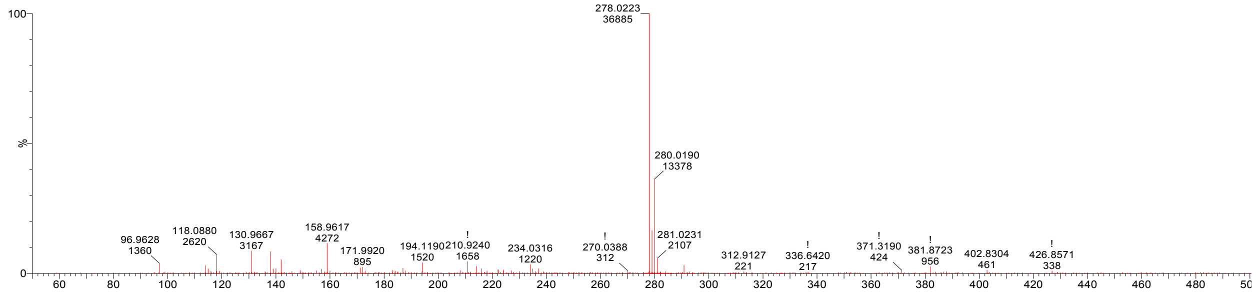
278.0223
36885
280.0190
13378
96.9628
1360
118.0880
2620
130.9667
3167
158.9617
4272
171.9920
895
194.1190
1520
210.9240
1658
234.0316
1220
270.0388
312
281.0231
2107
312.9127
221
336.6420
217
371.3190
424
381.8723
956
402.8304
461
426.8571
338
100
%
0
60
80
100
120
140
160
180
200
220
240
260
280
300
320
340
360
380
400
420
440
460
480
50

81

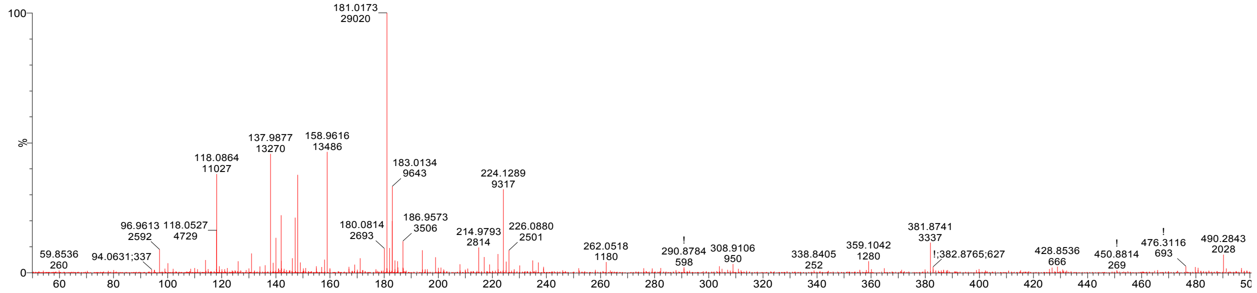
181.0173
29020
137.9877
13270
158.9616
13486
118.0864
11027
183.0134
9643
224.1289
9317
59.8536
260
94.0631;337
96.9613
2592
118.0527
4729
180.0814
2693
186.9573
3506
214.9793
2814
226.0880
2501
262.0518
1180
290.8784
598
308.9106
950
338.8405
252
359.1042
1280
381.8741
3337
!;382.8765;627
428.8536
666
450.8814
269
476.3116
693
490.2843
2028
100
%
0
60
80
100
120
140
160
180
200
220
240
260
280
300
320
340
360
380
400
420
440
460
480
50

82

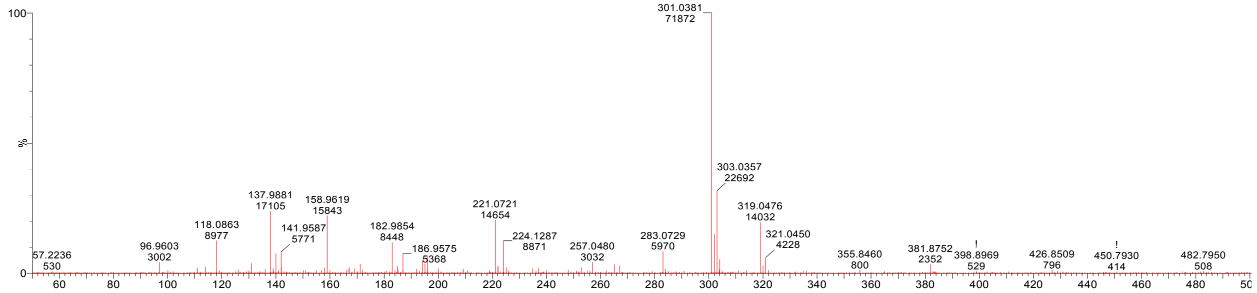
301.0381
71872
303.0357
22692
319.0476
14032
57.2236
530
96.9603
3002
118.0863
8977
137.9881
17105
141.9587
5771
158.9619
15843
182.9854
8448
186.9575
5368
221.0721
14654
224.1287
8871
257.0480
3032
283.0729
5970
321.0450
4228
355.8460
800
381.8752
2352
398.8969
529
426.8509
796
450.7930
414
482.7950
508
100
%
0
60
80
100
120
140
160
180
200
220
240
260
280
300
320
340
360
380
400
420
440
460
480
50

83

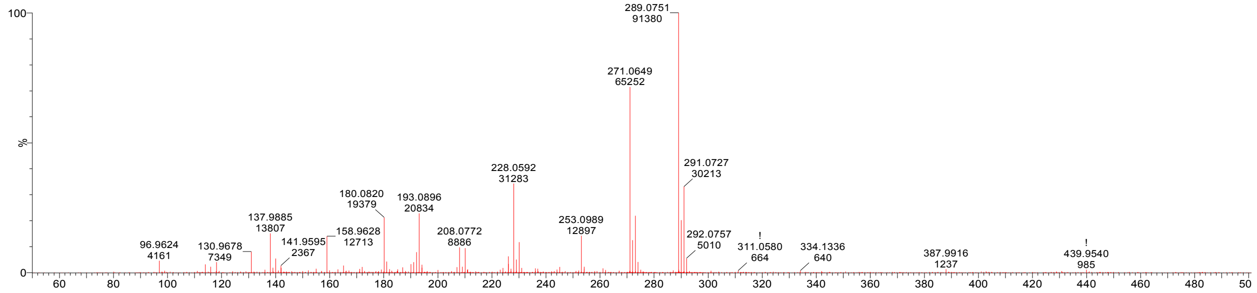
289.0751
91380
271.0649
65252
291.0727
30213
228.0592
31283
180.0820
19379
193.0896
20834
137.9885
13807
253.0989
12897
96.9624
4161
130.9678
7349
141.9595
2367
158.9628
12713
208.0772
8886
292.0757
5010
311.0580
664
334.1336
640
387.9916
1237
439.9540
985
100
%
0
60
80
100
120
140
160
180
200
220
240
260
280
300
320
340
360
380
400
420
440
460
480
50

84

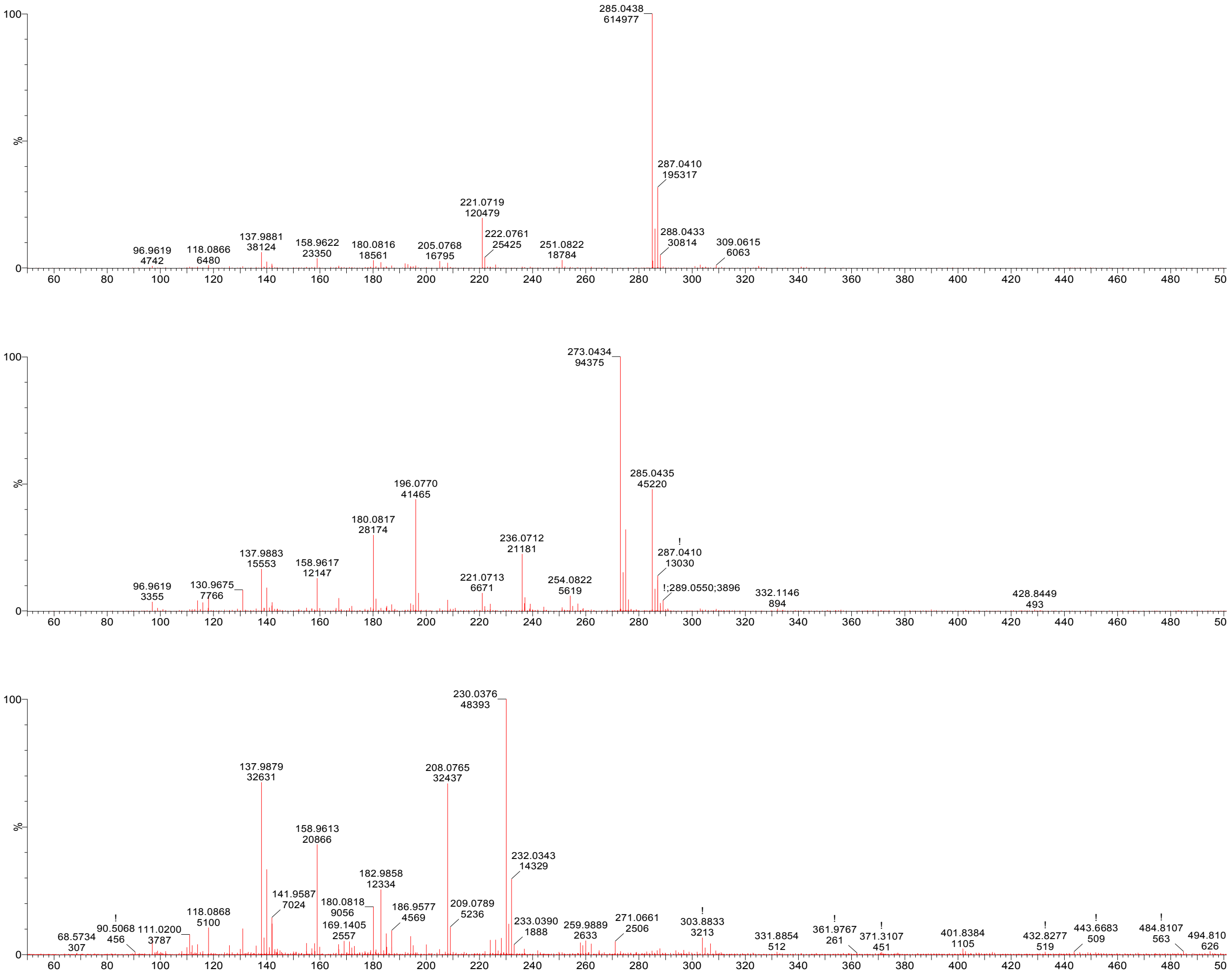

Figure S8. Mass spectra of TP278, TP181, TP301, TP289, TP285, TP273, and TP230.

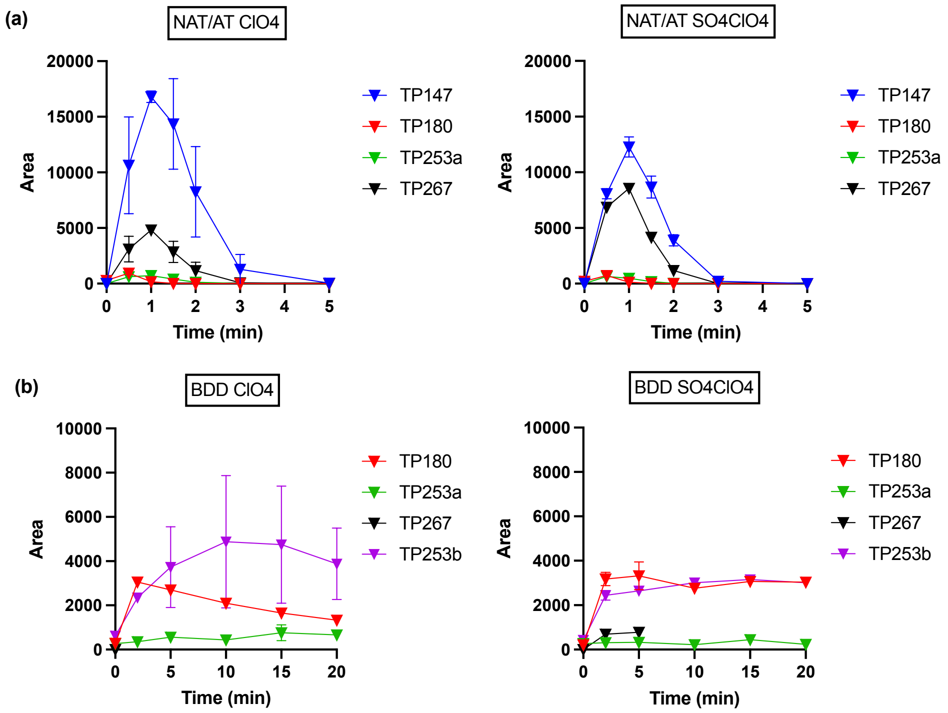

Figure S9. A zoomed-in look at CBZ transformation product evolution (excluding TP251) in $NaClO_4$ electrolytes at (a) NAT/AT and (b) BDD.

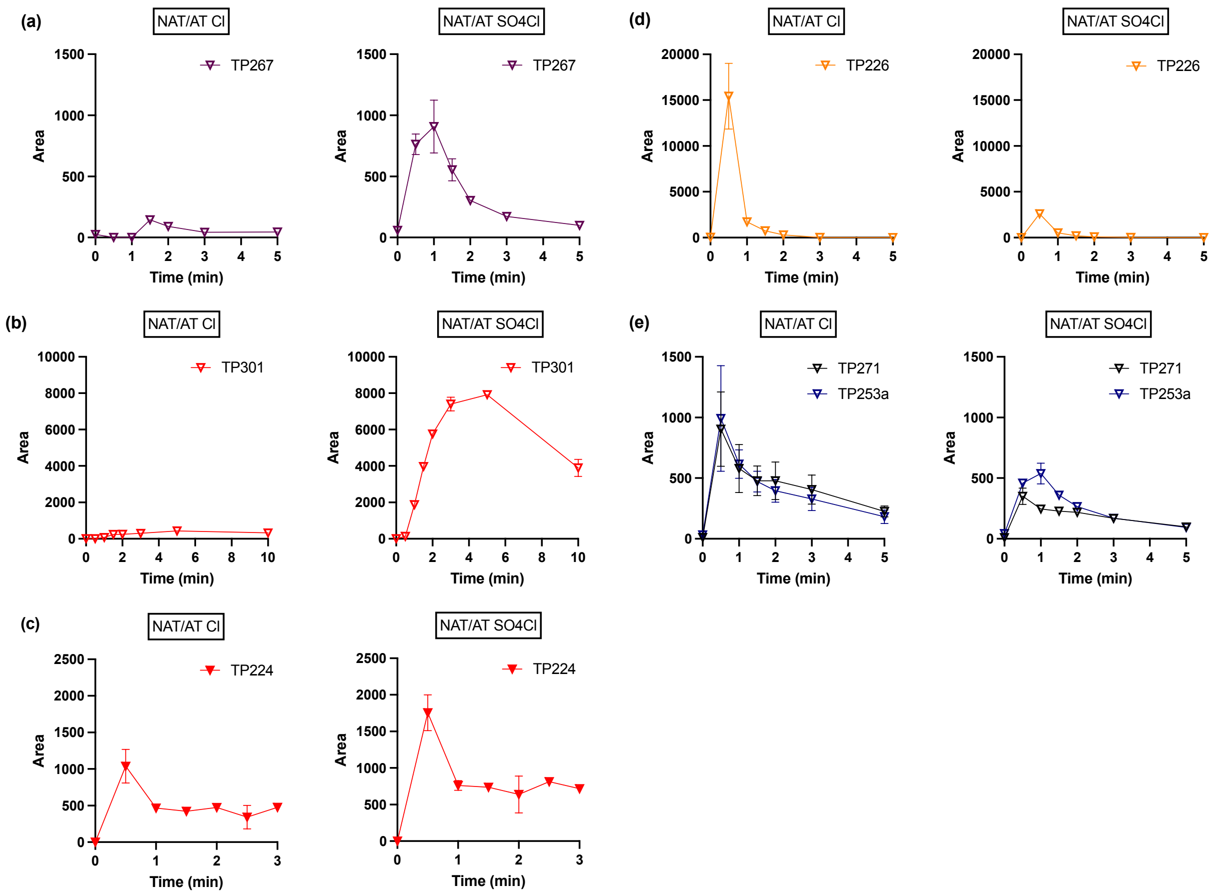

Figure S10. Responses of (a) TP267, (b) TP301, (c) TP224, (d) TP226, and (e) TP271 and TP253a of CBZ at NAT/AT in the absence (left) and presence (right) of 5 mM Na_2SO_4 in 50 mM NaCl electrolytes.

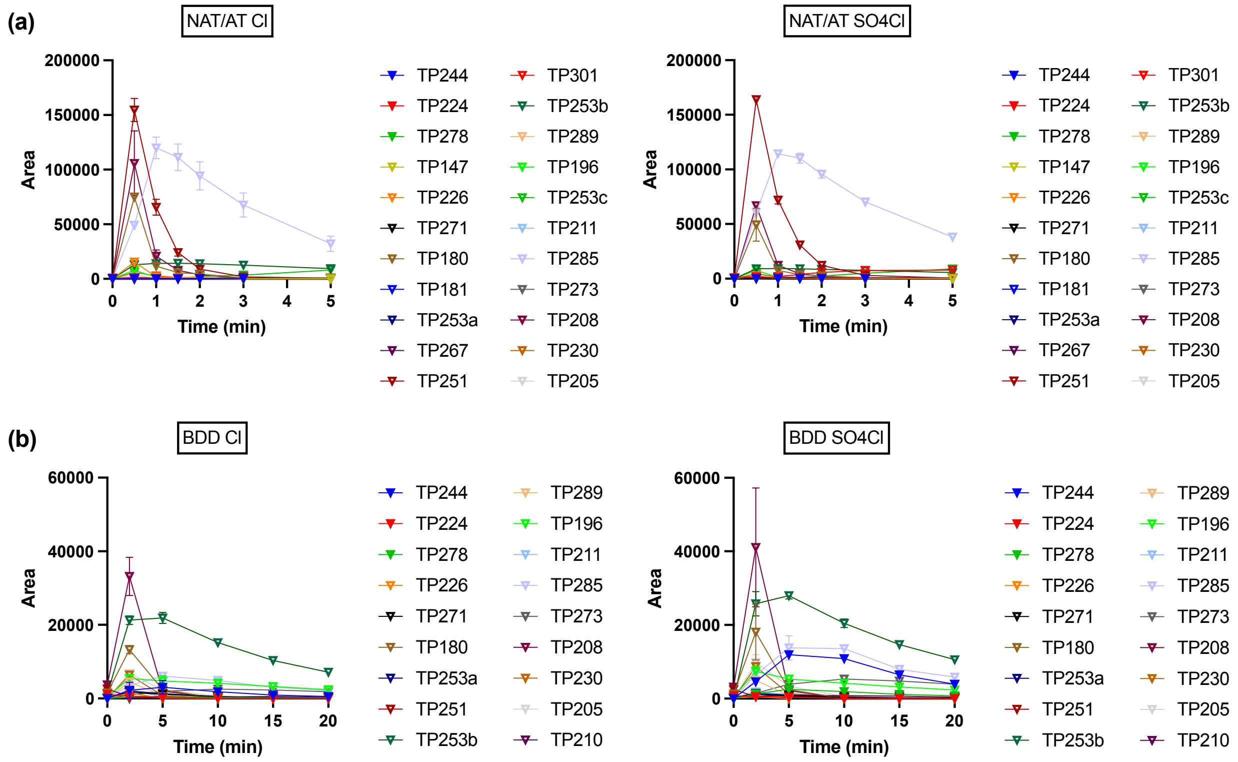

Figure S11. CBZ transformation product evolution (all) in NaCl electrolytes at (a) NAT/AT and (b) BDD.

Peak TP concentrations

	NAT/AT				BDD				SE[a]
	ClO_4	SO_4ClO_4	Cl	SO_4Cl	ClO_4	SO_4ClO_4	Cl	SO_4Cl	SE
**TP180, Acridine**									
Area 1	1104	835	73777	60103	3075	2967	14091	12716	273
Area 2	764	599	76140	38880	3033	3762	12485	23449	231
Avg	934	717	74959	49492	3054	3365	13288	18083	252
Conc (μM)	0.022	0.017	1.75	1.16	0.071	0.071	0.31	0.42	0.0059
**TP253b, Carbamazepine 10,11-epoxide**									
Area 1	1094	863	14918	9497	2762	3039	22940	27339	155
Area 2	1036	903	13519	8812	7003	3265	20827	28606	116
Avg	1065	883	14219	9155	4883	3152	21884	27973	136
Conc (μM)	0.076	0.063	1.01	0.65	0.35	0.22	1.55	1.99	0.0096
**TP224, 9-Acridinecarboxylic acid (estimated)[b]**									
Area 1	n.d.	n.d.	1200	1583	n.d.	n.d.	627	457	n.d.
Area 2	n.d.	n.d.	877	1929	n.d.	n.d.	387	570	n.d.
Avg	n.d.	n.d.	1039	1756	n.d.	n.d.	507	514	n.d.
Conc (μM)	n.d.	n.d.	0.032	0.054	n.d.	n.d.	0.015	0.016	n.d.

a. Treatment of secondary effluent by NAT/AT.
b. Estimated concentration only since exact hydrate number of the standard is not available.

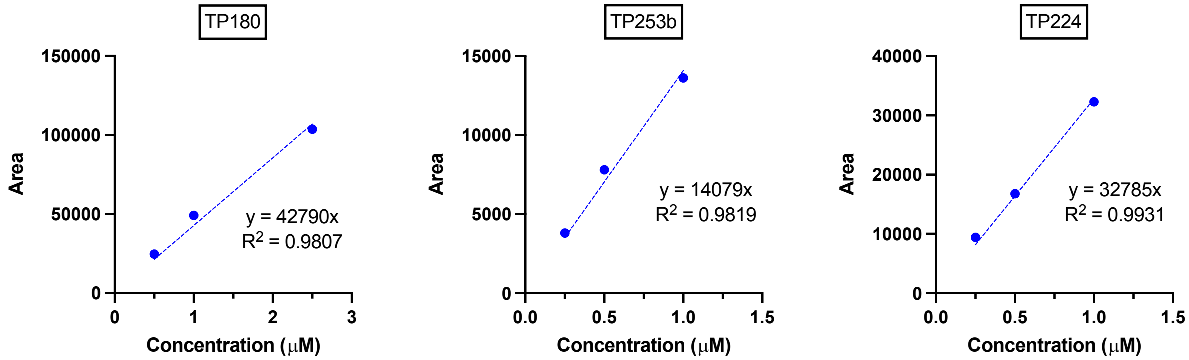

Figure S12. Peak concentrations and calibration curves of TP180 (acridine), TP253b (carbamazepine 10,11-epoxide), and TP224 (9-acridinecarboxylic acid) at NAT/AT and BDD in different electrolytes.

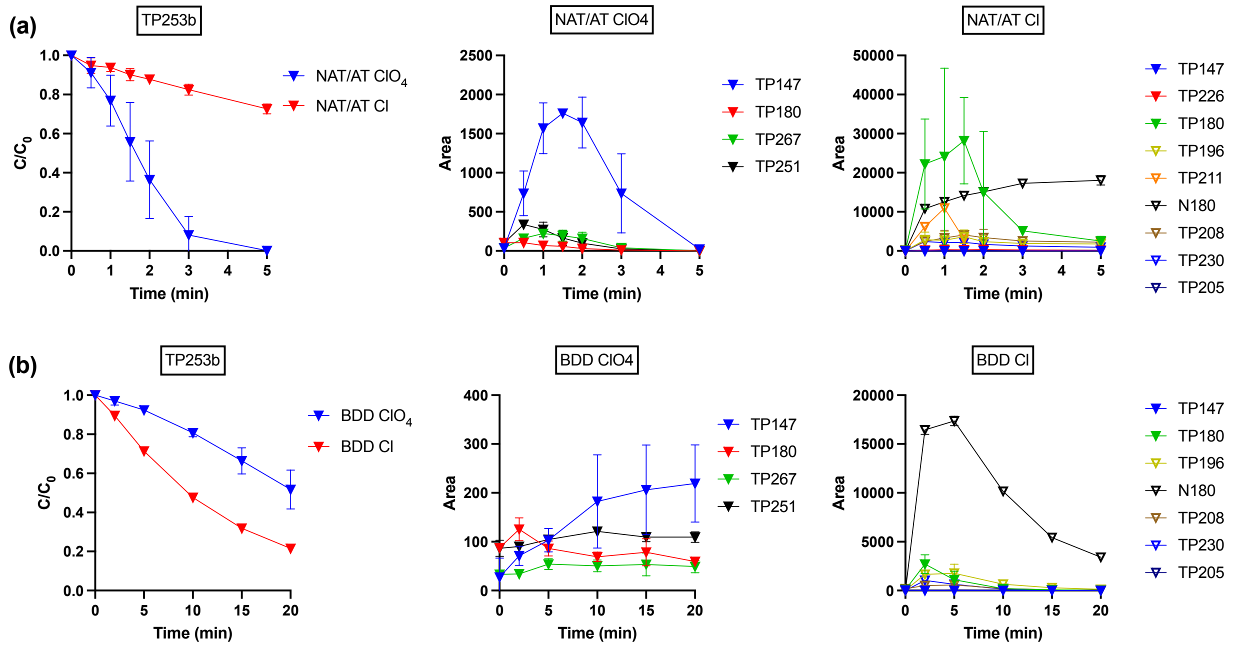

Figure S13. Electrochemical oxidation and transformation product evolution of 20 μM TP253b (CBZ-EP) at (a) NAT/AT and (b) BDD in 50 mM $NaClO_4$ and NaCl electrolytes.

Oxidation of CBZ to form TP251 (BQM) via TP253b (CBZ-EP) and TP271 (diOH-CBZ) has been proposed in the biodegradation with white-rot fungus *Pleurotus ostreatus*.[9,10] However, the intermediate TP271, which was commonly detected together with TP253b, was not detected in CBZ oxidation with both electrodes in $NaClO_4$ electrolytes. One possibility is that it was rapidly transformed.

To further investigate transformation pathways in the NAT/AT and BDD system, electrolysis of 20 μM TP253b (CBZ-EP) standard was conducted. It is obvious that O_3 greatly facilitated TP253b removal (Figure S13a, left). While both TP251 and TP147 were detected in $NaClO_4$ electrolytes, their reponses were very low comparing to those detected in CBZ degradation (Figure 4 and S9), especially considering the elevated initial TP253b concentration. The same was true for

the other two TPs, TP180 and TP267. Therefore, the above-proposed CBZ → TP253b → TP271 → TP251 pathway during biodegradation is likely not important to negligible in our system.

Similar observations can be made in NaCl electrolytes. While several CBZ TPs were deteced during TP253b oxidation, their responses were very low in comparison (Figure 5 and S11). However, the set of TPs (TP208, TP224, TP180, and TP196) in CBZ oxidation in NaCl electrolytes were all detected here as well, confirming the proposed TP253b → TP208 → TP224 → TP180 → TP196 sequence, even though it is not the only pathway. Other pathways likely contributed more significantly to the formation of these TPs during CBZ oxidation.

One other thing to note is that a new TP (N180, *m/z* = 180.0802, $C_{13}H_{10}N$) was detected. Its exact structure cannot be determined.

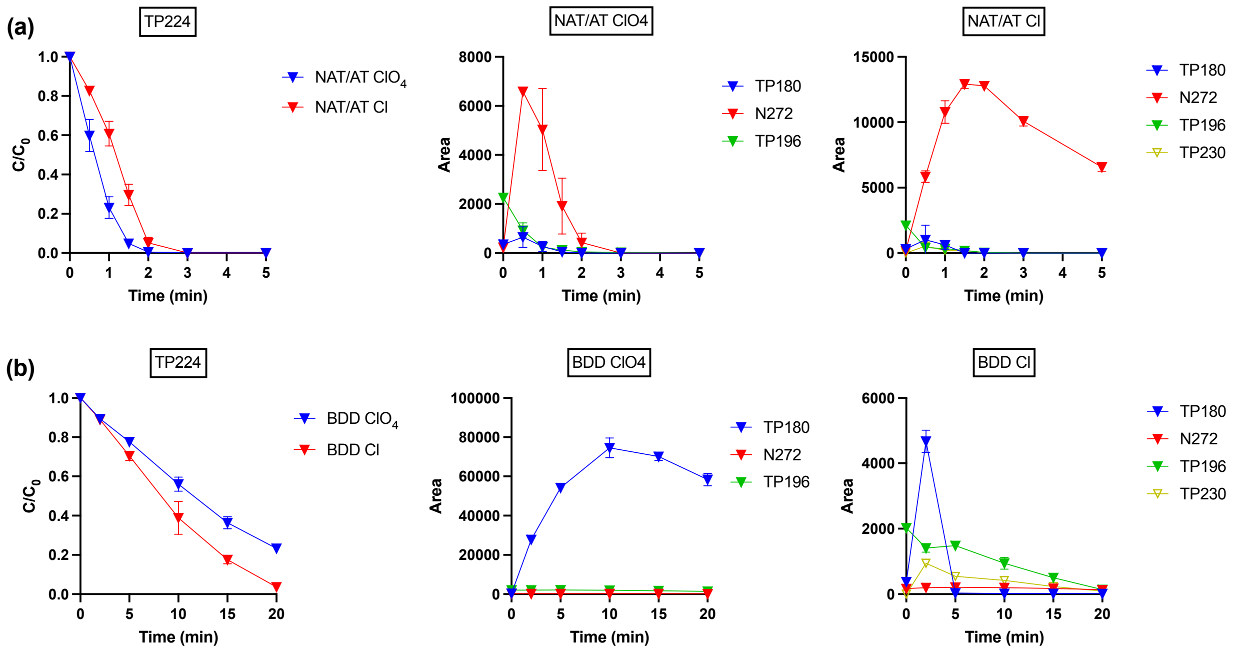

Figure S14. Electrochemical oxidation and transformation product evolution of 20 μM TP224 (9-acridinecarboxylic acid) at (a) NAT/AT and (b) BDD in 50 mM $NaClO_4$ and NaCl electrolytes.

It was observed that O_3 also promoted TP224 removal (Figure S14a, left). TP224 was only detected in NaCl electrolytes during CBZ degradation. Focusing on the two figures on the right (NAT/AT and BDD Cl), we can see that formation of TP180 and TP196 from TP224 was confirmed. TP230, the chlorinated product of TP196, was also detected. Similar to the case for TP253b, reponses of the detected TPs were very low comparing to those detected in CBZ degradation (Figure 5 and S11), considering the elevated initial TP224 concentration. Other pathways likely contributed more significantly to the formation of these TPs during CBZ oxidation.

A new TP (N272, *m/z* = 272.0558, $C_{14}H_{10}NO_5$) was detected. It is proposed to be the tri-hydroxylated product of TP224.

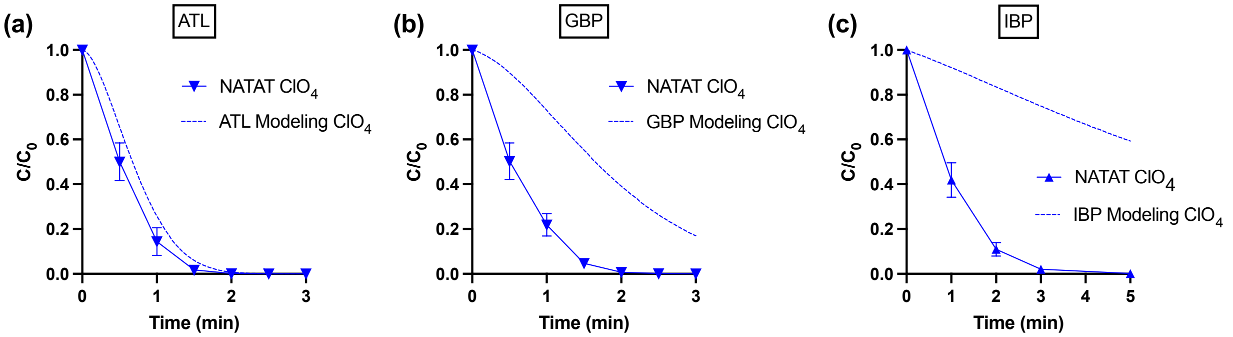

Figure S15. Experimental results and kinetic modeling predictions (dashed lines) for (a) ATL, (b) GBP, and (c) IBP degradation by NAT/AT in 50 mM $NaClO_4$ electrolytes.

Experimental degradation of both GBP (group II) and IBP (group III)[11,12] was faster than model predictions, suggesting accelerated kinetics through anodic O_3 activation.[13]

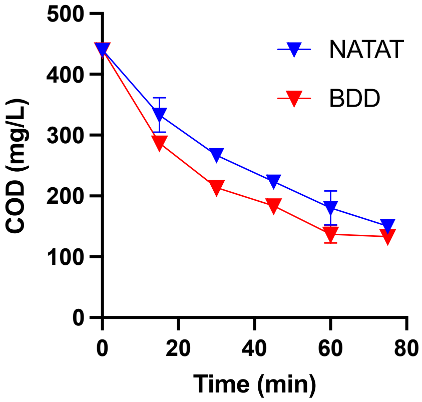

Figure S16. COD removal during treatment of latrine wastewater spiked with pharmaceuticals.

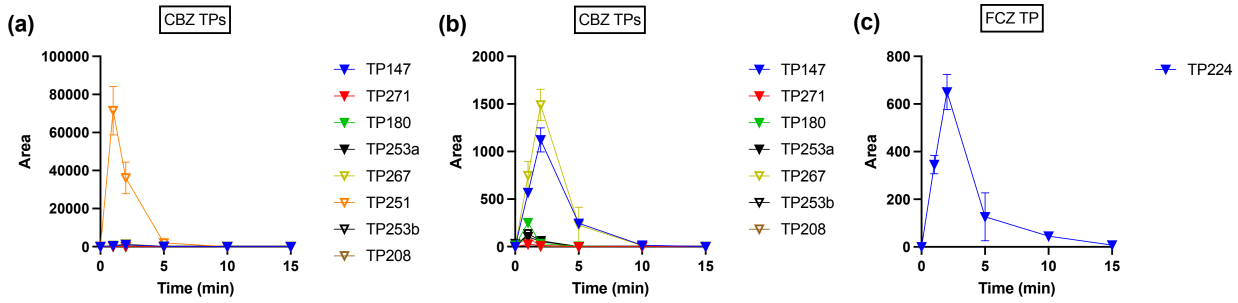

Figure S17. Transformation product evolution for (a) CBZ, (b) a zoomed-in look for CBZ excluding TP251, and (c) FCZ during treatment of secondary effluent spiked with pharmaceuticals by NAT/AT.

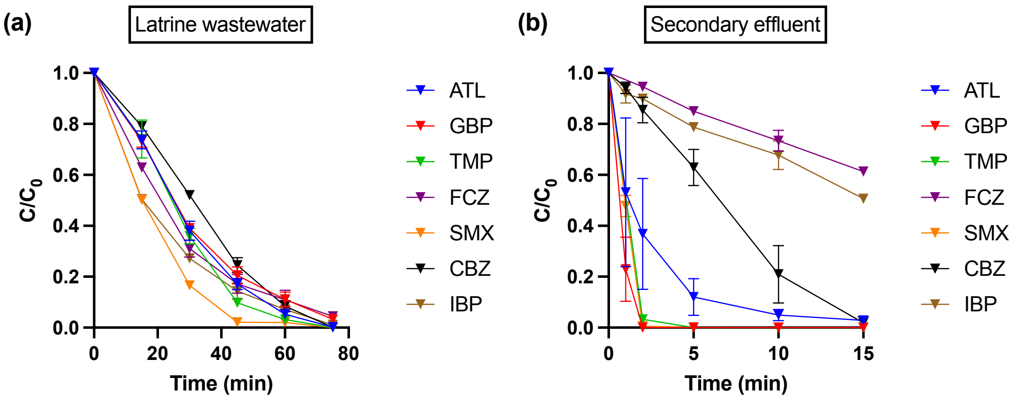

Figure S18. Removal of spiked pharmaceutical compounds (2 μM each) during electrolysis of (a) latrine wastewater and (b) secondary effluent by BDD.

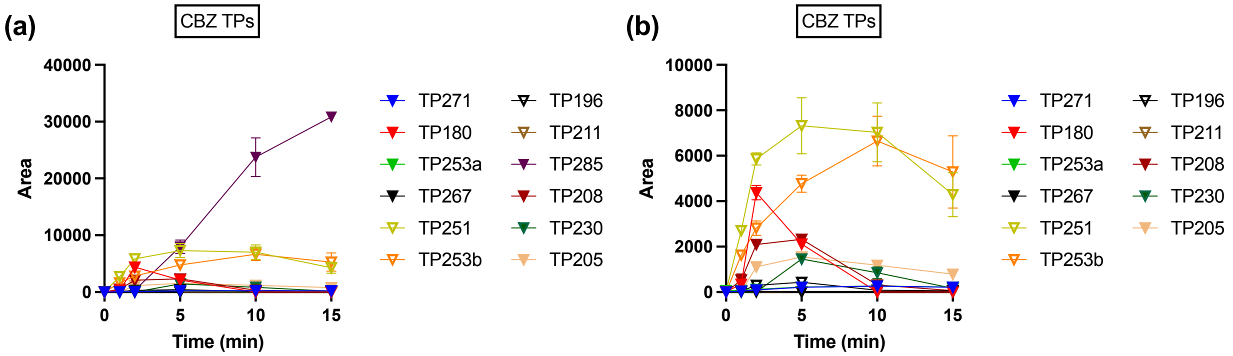

Figure S19. Transformation product evolution for (a) CBZ, and (b) a zoomed-in look for CBZ excluding TP285 during treatment of secondary effluent spiked with pharmaceuticals by BDD. TP224 of FCZ was not detected.

Text S1. Kinetic modeling.

Kinetic modeling was performed using the chemical kinetics software Kintecus 6.80.[14] A total of 117 elementary reactions were included in the model. pH was set to 2 in all fitting scenarios. Rate constants were obtained from literature. NAT/AT electrode-specific rate constants were fitted in a previous paper.[13] It is worth noting that the model can provide valuable insights and predictions for pharmaceutical degradation but in a relative sense, since reactive species generation (especially O_3) varies depending on pH and other factors such as $[Cl^-]$. In addition, in different solution matrices, reactive species are consumed to different degrees and sorption behaviors of organic compounds can also vary significantly.

Table S1. Principle reactions in the kinetic model.

No.	Reaction	Rate constant	Reference
1	$H^+ + OH^- \rightarrow H_2O$	1.00×10^{11} $M^{-1}s^{-1}$	[15]
2	$H_2O \rightarrow H^+ + OH^-$	1.00×10^{-3} s^{-1}	[15]
3	$H^+ + HO_2^- \rightarrow H_2O_2$	5.00×10^{10} $M^{-1}s^{-1}$	[15]
4	$H_2O_2 \rightarrow H^+ + HO_2^-$	1.30×10^{-1} s^{-1}	[15]
5	$H^+ + Cl^- \rightarrow HCl$	5.00×10^{10} $M^{-1}s^{-1}$	[15]
6	$HCl \rightarrow H^+ + Cl^-$	8.60×10^{16} s^{-1}	[15]
7	$H^+ + OCl^- \rightarrow HOCl$	5.00×10^{10} $M^{-1}s^{-1}$	[15]
8	$HOCl \rightarrow H^+ + OCl^-$	1.40×10^{3} s^{-1}	[15]
9	$H^+ + SO_4^{2-} \rightarrow HSO_4^-$	5.00×10^{10} $M^{-1}s^{-1}$	Assumed
10	$HSO_4^- \rightarrow H^+ + SO_4^{2-}$	6.00×10^{8} $M^{-1}s^{-1}$	Calculated[a]
11	$H^+ + SO_5^{2-} \rightarrow HSO_5^-$	5.00×10^{10} $M^{-1}s^{-1}$	[15]
12	$HSO_5^- \rightarrow H^+ + SO_5^{2-}$	2.00×10^{1} $M^{-1}s^{-1}$	[15]
13	$MO \rightarrow O_3$	$6.92/1.42 \times 10^{-7}$ s^{-1}	[13]
14	$MO \rightarrow HO\cdot$	1.69×10^{-6} s^{-1}	[13]

15	$MO + Cl^- \rightarrow Cl\cdot$	$1.50 \times 10^{-5}\ s^{-1}$	[13]
16	$MO + 2Cl^- \rightarrow Cl_2$	$8.05 \times 10^{-1}\ s^{-1}$	[13]
17	$MO + OCl^- \rightarrow OCl\cdot$	$3.89 \times 10^{2}\ s^{-1}$	[13]
18	$HO\cdot + H_2O_2 \rightarrow HO_2\cdot + H_2O$	$2.70 \times 10^{7}\ M^{-1}s^{-1}$	[16]
19	$HO\cdot + OH^- \rightarrow O\cdot^- + H_2O$	$1.20 \times 10^{10}\ M^{-1}s^{-1}$	[17]
20	$HO\cdot + HO\cdot \rightarrow H_2O_2$	$5.50 \times 10^{9}\ M^{-1}s^{-1}$	[16]
21	$HO\cdot + Cl^- \rightarrow ClOH\cdot^-$	$4.30 \times 10^{9}\ M^{-1}s^{-1}$	[18]
22	$HO\cdot \rightarrow$ Products	$1.00 \times 10^{7}\ s^{-1}$	[13]
23	$ClOH\cdot^- \rightarrow Cl^- + HO\cdot$	$6.10 \times 10^{9}\ s^{-1}$	[18]
24	$ClOH\cdot^- + H^+ \rightarrow Cl\cdot + H_2O$	$2.10 \times 10^{10}\ M^{-1}s^{-1}$	[18]
25	$ClOH\cdot^- + Cl^- \rightarrow Cl_2\cdot^- + OH^-$	$1.00 \times 10^{4}\ M^{-1}s^{-1}$	[19]
26	$Cl\cdot + H_2O \rightarrow ClOH\cdot^- + H^+$	$2.50 \times 10^{5}\ M^{-1}s^{-1}$	[20]
27	$Cl\cdot + H_2O_2 \rightarrow HO_2\cdot + Cl^- + H^+$	$2.00 \times 10^{9}\ M^{-1}s^{-1}$	[21]
28	$Cl\cdot + OH^- \rightarrow ClOH\cdot^-$	$1.80 \times 10^{10}\ M^{-1}s^{-1}$	[22]
29	$Cl\cdot + Cl^- \rightarrow Cl_2\cdot^-$	$8.00 \times 10^{9}\ M^{-1}s^{-1}$	[23]
30	$Cl_2\cdot^- + H_2O \rightarrow Cl^- + HClOH$	$1.30 \times 10^{3}\ M^{-1}s^{-1}$	[20]
31	$Cl_2\cdot^- + H_2O_2 \rightarrow HO_2\cdot + 2Cl^- + H^+$	$1.40 \times 10^{5}\ M^{-1}s^{-1}$	[24]
32	$Cl_2\cdot^- + OH^- \rightarrow ClOH\cdot^- + Cl^-$	$4.50 \times 10^{7}\ M^{-1}s^{-1}$	[19]
33	$Cl_2\cdot^- \rightarrow Cl\cdot + Cl^-$	$6.00 \times 10^{4}\ s^{-1}$	[23]
34	$HClOH \rightarrow Cl\cdot + H_2O$	$1.00 \times 10^{2}\ s^{-1}$	[20]
35	$HClOH \rightarrow ClOH\cdot^- + H^+$	$1.00 \times 10^{8}\ s^{-1}$	[20]
36	$HClOH + Cl^- \rightarrow Cl_2\cdot^- + H_2O$	$5.00 \times 10^{9}\ M^{-1}s^{-1}$	[20]
37	$Cl\cdot + Cl\cdot \rightarrow Cl_2$	$8.80 \times 10^{7}\ M^{-1}s^{-1}$	[25]
38	$Cl\cdot + Cl_2\cdot^- \rightarrow Cl^- + Cl_2$	$2.10 \times 10^{9}\ M^{-1}s^{-1}$	[21]
39	$Cl_2\cdot^- + HO\cdot \rightarrow HOCl + Cl^-$	$1.00 \times 10^{9}\ M^{-1}s^{-1}$	[26]
40	$Cl_2\cdot^- + Cl_2\cdot^- \rightarrow 2Cl^- + Cl_2$	$9.00 \times 10^{8}\ M^{-1}s^{-1}$	[21]

41	$Cl_2 + H_2O \rightarrow HOCl + Cl^- + H^+$	1.50×10^1 $M^{-1}s^{-1}$	27
42	$Cl_2 + H_2O_2 \rightarrow O_2 + 2HCl$	1.30×10^4 $M^{-1}s^{-1}$	24
43	$Cl_2 + O_2 \cdot^- \rightarrow O_2 + Cl_2 \cdot^-$	1.00×10^9 $M^{-1}s^{-1}$	24
44	$Cl_2 + HO_2 \cdot \rightarrow O_2 + Cl_2 \cdot^- + H^+$	1.00×10^9 $M^{-1}s^{-1}$	28
45	$Cl_2 + Cl^- \rightarrow Cl_3^-$	2.00×10^4 $M^{-1}s^{-1}$	29
46	$Cl_3^- + O_2 \cdot^- \rightarrow Cl_2 \cdot^- + Cl^- + O_2$	3.80×10^9 $M^{-1}s^{-1}$	24
47	$Cl_3^- + HO_2 \cdot \rightarrow Cl_2 \cdot^- + HCl + O_2$	1.00×10^9 $M^{-1}s^{-1}$	28
48	$Cl_3^- \rightarrow Cl_2 + Cl^-$	1.10×10^5 s^{-1}	29
49	$HOCl + H_2O_2 \rightarrow HCl + H_2O + O_2$	1.10×10^4 $M^{-1}s^{-1}$	30
50	$HOCl + HO \cdot \rightarrow OCl \cdot + H_2O$	2.00×10^9 $M^{-1}s^{-1}$	24
51	$HOCl + O_2 \cdot^- \rightarrow Cl \cdot + OH^- + O_2$	7.50×10^6 $M^{-1}s^{-1}$	24
52	$HOCl + HO_2 \cdot \rightarrow Cl \cdot + H_2O + O_2$	7.50×10^6 $M^{-1}s^{-1}$	24
53	$HOCl + Cl \cdot \rightarrow OCl \cdot + Cl^- + H^+$	3.00×10^9 $M^{-1}s^{-1}$	22
54	$HOCl + Cl^- + H^+ \rightarrow Cl_2 + H_2O$	1.82×10^4 $M^{-2}s^{-1}$	27
55	$OCl^- + H_2O_2 \rightarrow Cl^- + H_2O + O_2$	1.70×10^5 $M^{-1}s^{-1}$	30
56	$OCl^- + HO \cdot \rightarrow OCl \cdot + OH^-$	8.80×10^9 $M^{-1}s^{-1}$	24
57	$OCl^- + O_2 \cdot^- + H_2O \rightarrow Cl \cdot + 2OH^- + O_2$	2.00×10^8 $M^{-2}s^{-1}$	24
58	$OCl^- + Cl \cdot \rightarrow OCl \cdot + Cl^- + H^+$	8.20×10^9 $M^{-1}s^{-1}$	22
59	$OCl \cdot + OCl \cdot \rightarrow$ P3	7.50×10^9 $M^{-1}s^{-1}$	31
60	$HSO_4^- + HO \cdot \rightarrow SO_4 \cdot^- + H_2O$	6.90×10^5 $M^{-1}s^{-1}$	16
61	$HSO_4^- + O_3 \rightarrow$ Products	1.00×10^{-4} $M^{-1}s^{-1}$	32
62	$SO_4^{2-} + Cl \cdot \rightarrow SO_4 \cdot^- + Cl^-$	2.50×10^8 $M^{-1}s^{-1}$	33
63	$SO_4 \cdot^- + H_2O \rightarrow HSO_4^- + HO \cdot$	6.60×10^2 $M^{-1}s^{-1}$	34
64	$SO_4 \cdot^- + H_2O_2 \rightarrow HSO_4^- + HO_2 \cdot$	1.20×10^7 $M^{-1}s^{-1}$	35
65	$SO_4 \cdot^- + OH^- \rightarrow SO_4^{2-} + HO \cdot$	1.40×10^7 $M^{-1}s^{-1}$	34

66	$SO_4\cdot^- + HO\cdot \rightarrow HSO_5^-$	1.00×10^{10} $M^{-1}s^{-1}$	36
67	$SO_4\cdot^- + HO_2\cdot \rightarrow HSO_4^- + O_2$	3.50×10^{9} $M^{-1}s^{-1}$	37
68	$SO_4\cdot^- + SO_4\cdot^- \rightarrow S_2O_8^{2-}$	7.60×10^{8} $M^{-1}s^{-1}$	37
69	$SO_4\cdot^- + HSO_5^- \rightarrow HSO_4^- + SO_5\cdot^-$	1.00×10^{6} $M^{-1}s^{-1}$	38
70	$SO_4\cdot^- + SO_5^{2-} \rightarrow SO_4^{2-} + SO_5\cdot^-$	1.00×10^{8} $M^{-1}s^{-1}$	38
71	$SO_4\cdot^- + S_2O_8^{2-} \rightarrow SO_4^{2-} + S_2O_8\cdot^-$	6.60×10^{5} $M^{-1}s^{-1}$	37
72	$SO_4\cdot^- + Cl^- \rightarrow SO_4^{2-} + Cl\cdot$	4.70×10^{8} $M^{-1}s^{-1}$	33
73	$HSO_5^- + HO\cdot \rightarrow SO_5\cdot^- + H_2O$	1.70×10^{7} $M^{-1}s^{-1}$	39
74	$SO_5^{2-} + HO\cdot \rightarrow SO_5\cdot^- + OH^-$	2.10×10^{9} $M^{-1}s^{-1}$	39
75	$SO_5\cdot^- + HO_2\cdot \rightarrow HSO_5^- + O_2$	5.50×10^{7} $M^{-1}s^{-1}$	40
76	$SO_5\cdot^- + SO_5\cdot^- \rightarrow 2SO_4\cdot^- + O_2$	2.10×10^{8} $M^{-1}s^{-1}$	38
77	$SO_5\cdot^- + SO_5\cdot^- \rightarrow S_2O_8^{2-} + O_2$	2.20×10^{8} $M^{-1}s^{-1}$	38
78	$S_2O_8^{2-} + HO\cdot \rightarrow S_2O_8\cdot^- + OH^-$	1.40×10^{7} $M^{-1}s^{-1}$	41
79	$O_3 + H_2O_2 \rightarrow O_2 + HO\cdot + HO_2\cdot$	6.50×10^{-3} $M^{-1}s^{-1}$	42
80	$O_3 + OH^- \rightarrow O_2 + HO_2^-$	7.00×10^{1} $M^{-1}s^{-1}$	43
81	$O_3 + HO\cdot \rightarrow O_2 + HO_2\cdot$	1.10×10^{8} $M^{-1}s^{-1}$	43
82	$O_3 + O_2\cdot^- \rightarrow O_3\cdot^- + O_2$	1.60×10^{9} $M^{-1}s^{-1}$	44
83	$O_3 + HO_2^- \rightarrow O_2 + HO\cdot + O_2\cdot^-$	2.80×10^{6} $M^{-1}s^{-1}$	42
84	$O_3 + Cl^- \rightarrow O_2 + OCl^-$	3.00×10^{-3} $M^{-1}s^{-1}$	32
85	$O_3 + HOCl \rightarrow$ Products	2.00×10^{-3} $M^{-1}s^{-1}$	32
86	$O_3 + OCl^- \rightarrow$ Products	1.20×10^{2} $M^{-1}s^{-1}$	32
87	$O_3 + ClO_3^- \rightarrow$ Products	1.00×10^{-4} $M^{-1}s^{-1}$	32
88	$O_3 + ClO_4^- \rightarrow$ Products	2.00×10^{-5} $M^{-1}s^{-1}$	32
89	$O_3 + Cl_2\cdot^- \rightarrow$ Products	9.00×10^{7} $M^{-1}s^{-1}$	45
90	$O_3 \rightarrow$ Products	$7.04/3.28 \times 10^{-3}$ s^{-1}	13
91	$O_3\cdot^- + H^+ \rightarrow HO_3\cdot$	5.20×10^{10} $M^{-1}s^{-1}$	44
92	$O_3\cdot^- + HO\cdot \rightarrow HO_2\cdot + O_2\cdot^-$	8.50×10^{9} $M^{-1}s^{-1}$	46

93	$O_3^{\cdot -} + O^{\cdot -} \rightarrow 2O_2^{\cdot -}$	7.00×10^8 $M^{-1}s^{-1}$	[47]
94	$O_3^{\cdot -} \rightarrow O_2 + O^{\cdot -}$	3.30×10^3 s^{-1}	[48]
95	$O_2^{\cdot -} + H_2O_2 \rightarrow O_2 + HO\cdot + OH^-$	1.30×10^{-1} $M^{-1}s^{-1}$	[49]
96	$O_2^{\cdot -} + H^+ \rightarrow HO_2\cdot$	7.20×10^{10} $M^{-1}s^{-1}$	[50]
97	$O_2^{\cdot -} + HO\cdot \rightarrow O_2 + OH^-$	7.00×10^9 $M^{-1}s^{-1}$	[49]
98	$O_2^{\cdot -} + O^{\cdot -} + H_2O \rightarrow O_2 + 2OH^-$	6.00×10^8 $M^{-2}s^{-1}$	[47]
99	$O_2^{\cdot -} + HO_2\cdot \rightarrow HO_2^- + O_2$	9.70×10^7 $M^{-1}s^{-1}$	[49]
100	$O_2^{\cdot -} + Cl_2^{\cdot -} \rightarrow O_2 + 2Cl^-$	2.00×10^9 $M^{-1}s^{-1}$	[24]
101	$O_2^{\cdot -} + Cl^- \rightarrow$ Products	1.40×10^{-2} $M^{-1}s^{-1}$	[51]
102	$O_2^{\cdot -} + HOCl \rightarrow O_2 + Cl^- + HO\cdot$	7.50×10^6 $M^{-1}s^{-1}$	[51]
103	$O^{\cdot -} + O_2 \rightarrow O_3^{\cdot -}$	3.60×10^9 $M^{-1}s^{-1}$	[16]
104	$O^{\cdot -} + H_2O \rightarrow HO\cdot + OH^-$	1.70×10^6 $M^{-1}s^{-1}$	[16]
105	$O^{\cdot -} + HO\cdot \rightarrow HO_2^-$	2.00×10^{10} $M^{-1}s^{-1}$	[16]
106	$O^{\cdot -} + HO_2^- \rightarrow OH^- + O_2^{\cdot -}$	4.00×10^8 $M^{-1}s^{-1}$	[16]
107	$O^{\cdot -} + OCl^- \rightarrow OCl\cdot + OH^-$	2.30×10^8 $M^{-1}s^{-1}$	[16]
108	$HO_2\cdot + H_2O + O_2^{\cdot -} \rightarrow O_2 + H_2O_2 + OH^-$	9.70×10^7 $M^{-2}s^{-1}$	[51]
109	$HO_2\cdot + H_2O_2 \rightarrow O_2 + H_2O + HO\cdot$	3.00×10^0 $M^{-1}s^{-1}$	[49]
110	$HO_2\cdot + HO\cdot \rightarrow O_2 + H_2O$	6.60×10^9 $M^{-1}s^{-1}$	[49]
111	$HO_2\cdot + HO_2\cdot \rightarrow O_2 + H_2O_2$	8.30×10^5 $M^{-1}s^{-1}$	[49]
112	$HO_2\cdot + Cl_2^{\cdot -} \rightarrow O_2 + 2Cl^- + H^+$	3.00×10^9 $M^{-1}s^{-1}$	[24]
113	$HO_2\cdot + Cl_2 \rightarrow Cl_2^{\cdot -} + O_2 + H^+$	1.00×10^9 $M^{-1}s^{-1}$	[28]
114	$HO_2\cdot \rightarrow H^+ + O_2^{\cdot -}$	7.90×10^5 s^{-1}	[15]
115	$HO_2^- + HO\cdot \rightarrow HO_2\cdot + OH^-$	7.50×10^9 $M^{-1}s^{-1}$	[16]
116	$HO_3\cdot \rightarrow HO\cdot + O_2$	1.10×10^5 s^{-1}	[44]
117	$HO_3\cdot \rightarrow O_3^{\cdot -} + H^+$	3.70×10^4 s^{-1}	[44]

a. Calculated using the HSO_4^- acid dissociation constant $K_a = 1.2 \times 10^{-2}$ M (pKa = 1.92)[52]

Table S2. Fragmentation analyses of TP244, 278, 253a, 267, 253c, and 205 of CBZ.

TPs	Formula	Fragment *m/z*	Fragment elemental composition	Analysis
**TP244**	$C_{13}H_{10}NO_4$	200.0712<br>182.0611	$C_{12}H_{10}NO_2$<br>$C_{12}H_8NO$	- CO_2<br>- CO_2 - H_2O
**TP278**	$C_{13}H_9NO_4Cl$	234.0323<br>216.0220	$C_{12}H_9NO_2Cl$<br>$C_{12}H_7NOCl$	- CO_2<br>- CO_2 - H_2O
**TP253a**	$C_{15}H_{13}N_2O_2$	236.0711<br>210.0922<br>180.0817	$C_{15}H_{10}NO_2$<br>$C_{14}H_{12}NO$<br>$C_{13}H_{10}N$	- NH_3<br>- CHON<br>- NH_3 - 2CO
**TP267**	$C_{15}H_{11}N_2O_3$	249.0671<br>221.0719	$C_{15}H_9N_2O_2$<br>$C_{14}H_9N_2O$	- H_2O<br>- H_2O - CO
**TP253c**	$C_{14}H_9N_2O_3$	235.0508<br>179.0609	$C_{14}H_7N_2O_2$<br>$C_{12}H_7N_2$	- H_2O<br>- H_2O - 2CO
**TP205**	$C_{14}H_9N_2$	178.0656<br>151.0550	$C_{13}H_8N$<br>$C_{12}H_7$	- CHN<br>- 2CHN

Table S3. Sorted carbamazepine (CBZ) transformation product response[a] at NAT/AT and BDD.

$NaClO_4$ Electrolytes

TPs	NAT/AT ClO_4	TPs	NAT/AT SO_4ClO_4	TPs	BDD ClO_4	TPs	BDD SO_4ClO_4
TP251	0.90382989	TP251	0.96226418	TP251	0.98952151	TP251	0.99620415
TP147	0.03610589	TP147	0.03701393	TP253b	0.18088225	TP180	0.1227268
TP267	0.01011511	TP267	0.02578316	TP180	0.14145966	TP253b	0.10876842
TP180	0.00196006	TP180	0.00216116	TP253a	0.02828460	TP267	0.02676076
**TP253a**	0.00167781	TP253a	0.0019577	TP267	0.01201729	TP253a	0.01528693

NaCl Electrolytes

TPs	NAT/AT Cl	TPs	NAT/AT SO_4Cl	TPs	BDD Cl	TPs	BDD SO_4Cl
TP251	0.95408979	TP251	0.99613983	TP208	0.90001086	TP208	0.78266212
TP285	0.73981642	TP285	0.6952044	TP253b	0.59386958	TP253b	0.53289073
TP208	0.65311634	TP208	0.4061866	TP180	0.3606068	TP180	0.34448106
TP180	0.46270393	TP180	0.30062261	TP205	0.18332926	TP285	0.28327174
TP226	0.09525558	TP253b	0.05691551	TP230	0.17303048	TP244	0.22690124
TP253b	0.08776798	TP253c	0.05457997	TP285	0.16501126	TP205	0.18694277
TP253c	0.05010463	TP301	0.0481018	TP196	0.14764308	TP230	0.16624438
TP196	0.04887316	TP196	0.04570856	TP289	0.12194361	TP196	0.14185019
TP230	0.03736705	TP230	0.02646237	TP244	0.08163044	TP273	0.10105349

TP205	0.02609243	TP205	0.0177823	TP251	0.06925561	TP289	0.09438581
TP289	0.0134358	TP226	0.01577173	TP273	0.06841434	TP278	0.04815972
TP211	0.01318202	TP224	0.01087495	TP211	0.06335314	TP251	0.04692144
TP224	0.00792113	TP211	0.01051145	TP253a	0.05441125	TP211	0.03343367
TP253a	0.00612342	TP289	0.00872597	TP271	0.04591712	TP253a	0.02721367
TP271	0.0055833	TP181	0.00800583	TP226	0.03709734	TP271	0.02276537
TP244	0.00449258	TP278	0.00581835	TP224	0.02863036	TP210	0.01748838
TP301	0.0026327	TP267	0.00551236	TP210	0.01774811	TP224	0.01503086
TP273	0.00210493	TP253a	0.00327097	TP278	0.01287688	TP226	0.0089728
TP181	0.00146913	TP273	0.0021928				
TP147	0.00144135	TP271	0.00212902				
TP278	0.0013882	TP147	0.00083521				
TP267	0.00090123	TP244	0.00081129				

a. Take the peak area of the TP with highest maximum response in MS to be 1.0, the table summarized responses of other TPs relative to the max.
b. TPs marked with underline have, to the best of our knowledge, not been reported before.
c. TPs marked in green are not detected at BDD, and TPs marked in blue are not detected at NAT/AT, in respective electrolytes.

Table S4. Target pharmaceutical compound properties.

	**pKa**	**log K_{ow}**	**k_{O_3} ($M^{-1}s^{-1}$)[a]**	**Ref**	**$k_{\cdot OH}$ ($M^{-1}s^{-1}$)**	**Ref**
Carbamazepine (CBZ)	-	2.3-2.77	3.0×10^5	53	8.8×10^9	53
Fluconazole (FCZ)	2.6, 2.9, 11.0	0.25-0.5	2.0	11	4.4×10^9	11
Sulfamethoxazole (SMX)	5.7	0.89	5.7×10^5	54	5.5×10^9	53
Trimethoprim (TMP)	3.2, 7.1	0.91	2.7×10^5	54	6.9×10^9	54
Atenolol (ATL)	9.6	0.16	1.7×10^3	55	8.0×10^9	55
Gabapentin (GBP)	3.7	-1.1	2.2×10^2	11	9.1×10^9	11
Ibuprofen (IBP)	4.9	3.97	9.6	53	7.4×10^9	53

a. Rate constants with O_3 at pH 7.

Table S5. Composition of latrine wastewater and secondary effluent.

**Property**	**Latrine wastewater**	**Secondary effluent[a]**
pH	8.3	7.5
Conductivity (mS cm^{-1})	16.5	1.2

Cell voltage ($E_{WE} – E_{CE}$, V)	4.0 V (NAT/AT), 5.9 V (BDD)	5.4 V (NAT/AT), 7.5 V (BDD)
COD (mg O_2 L^{-1})	440	~80
[NH_4^+] (mM)	31	0.3
[Cl^-] (mM)	72	4.1
[Na^+] (mM)	81	6.0
[K^+] (mM)	-	0.4
[Mg^{2+}] (mM)	-	0.7

a. Composition of the secondary effluent was similar to a biologically-treated hospital wastewater used by Lan *et al.*[56] (pH 7.84, conductivity 1.2 mS/cm, COD 86 mg/L, [Cl^-] 2.0 mM, [Na^+] 7.0 mM, [K^+] 0.9 mM, [Mg^{2+}] 0.4 mM).

References

(1) Hübner, U.; Seiwert, B.; Reemtsma, T.; Jekel, M. Ozonation Products of Carbamazepine and Their Removal from Secondary Effluents by Soil Aquifer Treatment – Indications from Column Experiments. *Water Research* **2014**, *49*, 34–43.

(2) Martínez, C.; Canle L., M.; Fernández, M. I.; Santaballa, J. A.; Faria, J. Kinetics and Mechanism of Aqueous Degradation of Carbamazepine by Heterogeneous Photocatalysis Using Nanocrystalline TiO2, ZnO and Multi-Walled Carbon Nanotubes–Anatase Composites. *Applied Catalysis B: Environmental* **2011**, *102* (3), 563–571.

(3) Pan, Y.; Cheng, S.; Yang, X.; Ren, J.; Fang, J.; Shang, C.; Song, W.; Lian, L.; Zhang, X. UV/Chlorine Treatment of Carbamazepine: Transformation Products and Their Formation Kinetics. *Water Research* **2017**, *116*, 254–265.

(4) Wang, W.-L.; Wu, Q.-Y.; Huang, N.; Wang, T.; Hu, H.-Y. Synergistic Effect between UV and Chlorine (UV/Chlorine) on the Degradation of Carbamazepine: Influence Factors and Radical Species. *Water Research* **2016**, *98*, 190–198.

(5) Soufan, M.; Deborde, M.; Delmont, A.; Legube, B. Aqueous Chlorination of Carbamazepine: Kinetic Study and Transformation Product Identification. *Water Research* **2013**, *47* (14), 5076–5087.

(6) Keen, O. S.; Baik, S.; Linden, K. G.; Aga, D. S.; Love, N. G. Enhanced Biodegradation of Carbamazepine after UV/H2O2 Advanced Oxidation. *Environ. Sci. Technol.* **2012**, *46* (11), 6222–6227.

(7) Chiron, S.; Minero, C.; Vione, D. Photodegradation Processes of the Antiepileptic Drug Carbamazepine, Relevant To Estuarine Waters. *Environ. Sci. Technol.* **2006**, *40* (19), 5977–5983.

(8) Wu, Y.; Yang, Y.; Liu, Y.; Zhang, L.; Feng, L. Modelling Study on the Effects of Chloride on the Degradation of Bezafibrate and Carbamazepine in Sulfate Radical-Based Advanced Oxidation Processes: Conversion of Reactive Radicals. *Chemical Engineering Journal* **2019**, *358*, 1332–1341.

(9) Seiwert, B.; Golan-Rozen, N.; Weidauer, C.; Riemenschneider, C.; Chefetz, B.; Hadar, Y.; Reemtsma, T. Electrochemistry Combined with LC–HRMS: Elucidating Transformation

Products of the Recalcitrant Pharmaceutical Compound Carbamazepine Generated by the White-Rot Fungus Pleurotus Ostreatus. *Environ. Sci. Technol.* **2015**, *49* (20), 12342–12350.
(10) Golan-Rozen, N.; Seiwert, B.; Riemenschneider, C.; Reemtsma, T.; Chefetz, B.; Hadar, Y. Transformation Pathways of the Recalcitrant Pharmaceutical Compound Carbamazepine by the White-Rot Fungus Pleurotus Ostreatus: Effects of Growth Conditions. *Environ. Sci. Technol.* **2015**, *49* (20), 12351–12362.
(11) Lee, Y.; Kovalova, L.; McArdell, C. S.; von Gunten, U. Prediction of Micropollutant Elimination during Ozonation of a Hospital Wastewater Effluent. *Water Research* **2014**, *64*, 134–148.
(12) Lee, Y.; Gerrity, D.; Lee, M.; Bogeat, A. E.; Salhi, E.; Gamage, S.; Trenholm, R. A.; Wert, E. C.; Snyder, S. A.; von Gunten, U. Prediction of Micropollutant Elimination during Ozonation of Municipal Wastewater Effluents: Use of Kinetic and Water Specific Information. *Environ. Sci. Technol.* **2013**, *47* (11), 5872–5881.
(13) Zhang, Y.; Yang, Y.; Yang, S.; Quispe-Cardenas, E.; Hoffmann, M. R. Application of Heterojunction Ni–Sb–SnO2 Anodes for Electrochemical Water Treatment. *ACS EST Eng.* **2021**, *1* (8), 1236–1245.
(14) Ianni, J. C. *Kintecus*, Windows Version 6.80, 2020, www.kintecus.com.
(15) Yang, Y.; Pignatello, J. J.; Ma, J.; Mitch, W. A. Comparison of Halide Impacts on the Efficiency of Contaminant Degradation by Sulfate and Hydroxyl Radical-Based Advanced Oxidation Processes (AOPs). *Environ. Sci. Technol.* **2014**, *48* (4), 2344–2351.
(16) Buxton, G. V.; Greenstock, C. L.; Helman, W. P.; Ross, A. B. Critical Review of Rate Constants for Reactions of Hydrated Electrons, Hydrogen Atoms and Hydroxyl Radicals (·OH/·O $^{-}$ in Aqueous Solution. *Journal of Physical and Chemical Reference Data* **1988**, *17* (2), 513–886.
(17) V. Buxton, G. Pulse Radiolysis of Aqueous Solutions. Rate of Reaction of OH with OH –. *Transactions of the Faraday Society* **1970**, *66* (0), 1656–1660.
(18) G. Jayson, G.; J. Parsons, B.; J. Swallow, A. Some Simple, Highly Reactive, Inorganic Chlorine Derivatives in Aqueous Solution. Their Formation Using Pulses of Radiation and Their Role in the Mechanism of the Fricke Dosimeter. *Journal of the Chemical Society, Faraday Transactions 1: Physical Chemistry in Condensed Phases* **1973**, *69* (0), 1597–1607.
(19) Grigor'ev, A. E.; Makarov, I. E.; Pikaev, A. K. Formation of Cl_2^- in the bulk of solution during radiolysis of concentrated aqueous solutions of chlorides. *Khimiya Vysokikh Ehnergij* **1987**, *21* (2), 123–126.
(20) McElroy, W. John. A Laser Photolysis Study of the Reaction of Sulfate(1-) with Chloride and the Subsequent Decay of Chlorine(1-) in Aqueous Solution. *The Journal of Physical Chemistry* **1990**, *94* (6), 2435–2441.
(21) Yu, X.-Y.; Barker, J. R. Hydrogen Peroxide Photolysis in Acidic Aqueous Solutions Containing Chloride Ions. II. Quantum Yield of HO•(Aq) Radicals. *J. Phys. Chem. A* **2003**, *107* (9), 1325–1332.
(22) Kläning, U. K.; Wolff, T. Laser Flash Photolysis of HClO, ClO−, HBrO, and BrO− in Aqueous Solution. Reactions of Cl- and Br-Atoms. *Berichte der Bunsengesellschaft für physikalische Chemie* **1985**, *89* (3), 243–245.
(23) Nagarajan, V.; Fessenden, R. W. Flash Photolysis of Transient Radicals. 1. X2- with X = Cl, Br, I, and SCN. *The Journal of Physical Chemistry* **1985**, *89* (11), 2330–2335.

(24) Matthew, B. M.; Anastasio, C. A Chemical Probe Technique for the Determination of Reactive Halogen Species in Aqueous Solution: Part 1 ? Bromide Solutions. *Atmospheric Chemistry and Physics Discussions* **2006**, *6* (1), 899–940.
(25) Wu, D.; Wong, D.; Di Bartolo, B. Evolution of Cl−2 in Aqueous NaCl Solutions. *Journal of Photochemistry* **1980**, *14* (4), 303–310.
(26) Wagner, I.; Karthäuser, J.; Strehlow, H. On the Decay of the Dichloride Anion Cl−2 in Aqueous Solution. *Berichte der Bunsengesellschaft für physikalische Chemie* **1986**, *90* (10), 861–867.
(27) Wang, T. X.; Margerum, D. W. Kinetics of Reversible Chlorine Hydrolysis: Temperature Dependence and General-Acid/Base-Assisted Mechanisms. *Inorganic Chemistry* **1994**, *33* (6), 1050–1055.
(28) Bjergbakke, E.; Navaratnam, S.; Parsons, B. J.; Swallow, A. J. Reaction between Hydroperoxo Radicals and Chlorine in Aqueous Solution. *Journal of the American Chemical Society* **1981**, *103* (19), 5926–5928.
(29) Ershov, B. G. Kinetics, Mechanism and Intermediates of Some Radiation-Induced Reactions in Aqueous Solutions. *Russ. Chem. Rev.* **2004**, *73* (1), 101–113.
(30) Connick, R. E. The Interaction of Hydrogen Peroxide and Hypochlorous Acid in Acidic Solutions Containing Chloride Ion. *Journal of the American Chemical Society* **1947**, *69* (6), 1509–1514.
(31) V. Buxton, G.; S. Subhani, M. Radiation Chemistry and Photochemistry of Oxychlorine Ions. Part 1.—Radiolysis of Aqueous Solutions of Hypochlorite and Chlorite Ions. *Journal of the Chemical Society, Faraday Transactions 1: Physical Chemistry in Condensed Phases* **1972**, *68* (0), 947–957.
(32) Hoigné, J.; Bader, H.; Haag, W. R.; Staehelin, J. Rate Constants of Reactions of Ozone with Organic and Inorganic Compounds in Water—III. Inorganic Compounds and Radicals. *Water Research* **1985**, *19* (8), 993–1004.
(33) Huie, R. E.; Clifton, C. L.; Neta, P. Electron Transfer Reaction Rates and Equilibria of the Carbonate and Sulfate Radical Anions. *International Journal of Radiation Applications and Instrumentation. Part C. Radiation Physics and Chemistry* **1991**, *38* (5), 477–481.
(34) Herrmann, H.; Reese, A.; Zellner, R. Time-Resolved UV/VIS Diode Array Absorption Spectroscopy of SOx−(X=3, 4, 5) Radical Anions in Aqueous Solution. *Journal of Molecular Structure* **1995**, *348*, 183–186.
(35) Wine, P. H.; Tang, Y.; Thorn, R. P.; Wells, J. R.; Davis, D. D. Kinetics of Aqueous Phase Reactions of the SO4 − Radical with Potential Importance in Cloud Chemistry. *Journal of Geophysical Research: Atmospheres* **1989**, *94* (D1), 1085–1094.
(36) Klaning, U. K.; Sehested, K.; Appelman, E. H. Laser Flash Photolysis and Pulse Radiolysis of Aqueous Solutions of the Fluoroxysulfate Ion, SO4F-. *Inorg. Chem.* **1991**, *30* (18), 3582–3584.
(37) Jiang, P.-Y.; Katsumura, Y.; Nagaishi, R.; Domae, M.; Ishikawa, K.; Ishigure, K.; Yoshida, Y. Pulse Radiolysis Study of Concentrated Sulfuric Acid Solutions. Formation Mechanism, Yield and Reactivity of Sulfate Radicals. *J. Chem. Soc., Faraday Trans.* **1992**, *88* (12), 1653–1658.
(38) Das, T. N. Reactivity and Role of SO5•- Radical in Aqueous Medium Chain Oxidation of Sulfite to Sulfate and Atmospheric Sulfuric Acid Generation. *J. Phys. Chem. A* **2001**, *105* (40), 9142–9155.

(39) Maruthamuthu, P.; Neta, P. Radiolytic Chain Decomposition of Peroxomonophosphoric and Peroxomonosulfuric Acids. *J. Phys. Chem.* **1977**, *81* (10), 937–940.
(40) Yermakov, A. N.; Zhitomirsky, B. M.; Poskrebyshev, G. A.; Stoliarov, S. I. Kinetic Study of SO5- and HO2 Radicals Reactivity in Aqueous Phase Bisulfite Oxidation. *J. Phys. Chem.* **1995**, *99* (10), 3120–3127.
(41) Buxton, G. V.; Salmon, G. A.; Wood, N. D. A Pulse Radiolysis Study of the Chemistry of Oxysulphur Radicals in Aqueous Solution. In *Physico-Chemical Behaviour of Atmospheric Pollutants: Air Pollution Research Reports*; Restelli, G., Angeletti, G., Eds.; Springer Netherlands: Dordrecht, 1990; pp 245–250.
(42) Staehelin, Johannes.; Hoigne, Juerg. Decomposition of Ozone in Water: Rate of Initiation by Hydroxide Ions and Hydrogen Peroxide. *Environ. Sci. Technol.* **1982**, *16* (10), 676–681.
(43) Lesko, T. M.; Colussi, A. J.; Hoffmann, M. R. Hydrogen Isotope Effects and Mechanism of Aqueous Ozone and Peroxone Decompositions. *J. Am. Chem. Soc.* **2004**, *126* (13), 4432–4436.
(44) Buehler, R. E.; Staehelin, J.; Hoigne, J. Ozone Decomposition in Water Studied by Pulse Radiolysis. 1. Perhydroxyl (HO2)/Hyperoxide (O2-) and HO3/O3- as Intermediates. *J. Phys. Chem.* **1984**, *88* (12), 2560–2564.
(45) Bielski, B. H. J. A Pulse Radiolysis Study of the Reaction of Ozone with Cl∸2 in Aqueous Solutions. *Radiation Physics and Chemistry* **1993**, *41* (3), 527–530.
(46) Sehested, K.; Holcman, J.; Bjergbakke, E.; Hart, E. J. Formation of Ozone in the Reaction of Hydroxyl with O3- and the Decay of the Ozonide Ion Radical at PH 10-13. *The Journal of Physical Chemistry* **1984**, *88* (2), 269–273.
(47) Sehested, K.; Holcman, J.; Bjergbakke, E.; Hart, E. J. Ultraviolet Spectrum and Decay of the Ozonide Ion Radical, O3-, in Strong Alkaline Solution. *The Journal of Physical Chemistry* **1982**, *86* (11), 2066–2069.
(48) Gall, B. L.; Dorfman, L. M. Pulse Radiolysis Studies. XV. Reactivity of the Oxide Radical Ion and of the Ozonide Ion in Aqueous Solution. *Journal of the American Chemical Society* **1969**, *91* (9), 2199–2204.
(49) Crittenden, J. C.; Hu, S.; Hand, D. W.; Green, S. A. A Kinetic Model for H2O2/UV Process in a Completely Mixed Batch Reactor. *Water Research* **1999**, *33* (10), 2315–2328.
(50) Field, R. J.; Noyes, R. M.; Postlethwaite, D. Photoreduction of Hydrogen Peroxide by Hydrogen. *The Journal of Physical Chemistry* **1976**, *80* (3), 223–229.
(51) Bielski, B. H. J.; Cabelli, D. E.; Arudi, R. L.; Ross, A. B. Reactivity of HO2/O−2 Radicals in Aqueous Solution. *Journal of Physical and Chemical Reference Data* **1985**, *14* (4), 1041–1100.
(52) McMurry, J. E.; Fay, R. C. *General Chemistry: Atoms First*; Pearson Higher Ed: Upper Saddle River, NJ, 2010.
(53) Huber, M. M.; Canonica, S.; Park, G.-Y.; von Gunten, U. Oxidation of Pharmaceuticals during Ozonation and Advanced Oxidation Processes. *Environ. Sci. Technol.* **2003**, *37* (5), 1016–1024.
(54) Dodd, M. C.; Buffle, M.-O.; von Gunten, U. Oxidation of Antibacterial Molecules by Aqueous Ozone: Moiety-Specific Reaction Kinetics and Application to Ozone-Based Wastewater Treatment. *Environ. Sci. Technol.* **2006**, *40* (6), 1969–1977.
(55) Benner, J.; Salhi, E.; Ternes, T.; von Gunten, U. Ozonation of Reverse Osmosis Concentrate: Kinetics and Efficiency of Beta Blocker Oxidation. *Water Research* **2008**, *42* (12), 3003–3012.

(56) Lan, Y.; Coetsier, C.; Causserand, C.; Groenen Serrano, K. On the Role of Salts for the Treatment of Wastewaters Containing Pharmaceuticals by Electrochemical Oxidation Using a Boron Doped Diamond Anode. *Electrochimica Acta* **2017**, *231*, 309–318.
